# The Importance of the Human Footprint in Shaping the Global Distribution of Terrestrial, Freshwater and Marine Invaders

**DOI:** 10.1371/journal.pone.0125801

**Published:** 2015-05-27

**Authors:** Belinda Gallardo, Alexandra Zieritz, David C. Aldridge

**Affiliations:** 1 Aquatic Ecology Group, Department of Zoology, Cambridge University, Cambridge, United Kingdom; 2 Applied and Restoration Ecology Group, Department of Biodiversity and Restoration, Pyrenean Institute of Ecology (IPE-CSIC), Zaragoza, Spain; College of Charleston, UNITED STATES

## Abstract

Human activities such as transport, trade and tourism are likely to influence the spatial distribution of non-native species and yet, Species Distribution Models (SDMs) that aim to predict the future broad scale distribution of invaders often rely on environmental (e.g. climatic) information only. This study investigates if and to what extent do human activities that directly or indirectly influence nature (hereafter the human footprint) affect the global distribution of invasive species in terrestrial, freshwater and marine ecosystems. We selected 72 species including terrestrial plants, terrestrial animals, freshwater and marine invasive species of concern in a focus area located in NW Europe (encompassing Great Britain, France, The Netherlands and Belgium). Species Distribution Models were calibrated with the global occurrence of species and a set of high-resolution (9×9 km) environmental (e.g. topography, climate, geology) layers and human footprint proxies (e.g. the human influence index, population density, road proximity). Our analyses suggest that the global occurrence of a wide range of invaders is primarily limited by climate. Temperature tolerance was the most important factor and explained on average 42% of species distribution. Nevertheless, factors related to the human footprint explained a substantial amount (23% on average) of species distributions. When global models were projected into the focus area, spatial predictions integrating the human footprint featured the highest cumulative risk scores close to transport networks (proxy for invasion pathways) and in habitats with a high human influence index (proxy for propagule pressure). We conclude that human related information–currently available in the form of easily accessible maps and databases—should be routinely implemented into predictive frameworks to inform upon policies to prevent and manage invasions. Otherwise we might be seriously underestimating the species and areas under highest risk of future invasions.

## Introduction

Species Distribution Models (SDMs) are increasingly used to investigate spatial patterns in the distribution of species [1], including invasive ones [2]. In brief, SDMs correlate the occurrence of a given species with the environmental conditions of the sites it inhabits in order to locate areas that are most similar to its current distribution, and thus likely to be the most susceptible to invasion in the future [3]. Predictive SDMs have traditionally been calibrated with bioclimatic factors that are known to constrain species distribution at regional to global scales [4]. For instance, temperature affects body size, reproduction, growth and survival, and is consequently a key factor in determining the success of non-native species during different stages of the invasion process [5]. However, non-native species often exhibit an extraordinary ability for adaptation and expand their distribution towards new climates [6]. Climatic factors alone may thus be insufficient for accurate prediction of the potential distribution of invasive species [7–9].

The most important non-climatic factors that are likely to play a significant role in the spatial distribution of invaders are those related to human activities. Trade, transport, travel, and tourism amongst other anthropogenic factors have consistently been related to the rapid increase in the number and impact of invasive species globally [10, 11]. For instance, ports are the main entrance point of non-natives from other continents arriving as imports (e.g. plants and animals), contaminants of products (e.g. timber pathogens) or stowaways (e.g. ship hull fouling or transport with ballast water) [12]. Roads, railways and canals provide pathways along which species can disperse (e.g. [13, 14]). Human population density and wealth are associated with habitat degradation, as well as high rates of invasion (e.g. [10, 15, 16]). Consequently, agricultural, urban and industrial landscapes usually attain high levels of invasion (e.g. [15, 17]). The combination of human activities that directly or indirectly influence nature is considered in the present study as the “human footprint” [18].

While the relationship between invasive species and human activities has been widely explored, the majority of studies have been conducted at the local scale. Studies that have investigated patterns on broad, continental scales have often been based on data sets at the regional or country level (e.g. richness of invasive species per country or state [10, 19]), due to detailed data on human activities at the global scale being unavailable at that time [18]. However, recent advances in satellite imagery and geographic information systems together with improved reporting of population statistics have enabled the development of global scale indicators of the human footprint [18]. Taking advantage of this new source of information, a recent study demonstrated that climate based models underestimated by a 20% the risk of freshwater invasion when compared to models integrating human indicators such as population density, human degradation and port proximity [20]. If and to which extent the human footprint affects the spatial distribution of other groups of invaders and habitats remains to be tested, however. Consequently, despite their potential to improve considerably predictions on future invasions, human activities have yet to be integrated routinely into SDMs to inform policies for preventing and managing invasions [9, 10, 20].

This study investigates the relative influence of environmental and anthropogenic factors (the human footprint) on the global distribution of invasive species. Using SDM, we modelled the distribution of 72 invasive species covering a broad spectrum of life forms and habitats: terrestrial plants, terrestrial animals, freshwater and marine organisms. This set represents some of the species with the highest impact on biodiversity and socio-economic activities in Europe. We expected climatic variables to set the basic limits for the global distribution of species, and human footprint to promote the dispersal and establishment of invasive species in suitable geographic areas [20]. Finally, globally calibrated models were appraised within a smaller focus area to evaluate spatial predictions derived from SDM integrating the human footprint. This multi-scale approach allowed investigating i) the response of invasive species to global environmental and human related processes, and ii) the ability of human footprint-inclusive SDMs to locate potential hotspots of invasion at the regional scale. By using geographical information at a relatively high resolution and broad scale, this study provides accurate insights into the spatial correlation between invasive species occurrence and multiple environmental and human related indicators at a global scale. Furthermore, the multispecies approach enabled the comparison of patterns between habitats and species groups that are otherwise difficult to contrast when modelling habitats separately.

## Methods

### Focus area

Models developed in this study utilize a global coverage. However, we selected a focus area to illustrate the ability of human footprint-inclusive SDMs to inform management decisions at regional scale. The focus area encompasses four countries—Great Britain, France, Belgium and The Netherlands—including the British Channel and the southern part of the North Sea. The region has a long history of trade and travel, and includes important commercial ports. These intensive activities across national borders have led to the introduction of over 6,000 non-native animal, plant and other species to this area, both from other European regions and further afield [21]. The focus area affected the selection of species for modelling and the interpretation of regional spatial patterns of invasion.

#### Selection of invasive species for modelling

To ensure maximum relevance to policy makers, we selected those invasive species for modelling that would potentially cause the greatest ecological and socio-economic harm in the four countries under investigation (i.e. Great Britain, France, Belgium and The Netherlands). This was done in two steps. Firstly, we compiled a list of 340 of the ‘worst’ current and potential invasive species identified in previous international, national and regional horizon scanning implementations (data sources summarized in S1 Table).

In a second step, from the preliminary list we selected 72 species including 17 terrestrial plants, 19 terrestrial animals, 17 freshwater and 19 marine organisms (S2 Table). The selection was based on the availability of reliable data for modelling, the risk of establishment and the impacts associated to the species. The final selection of species was dominated by organisms belonging to the phyla Angiospermae (28%), followed by Arthropoda (26%) and Chordata (25%) (more information in S2 Table). This list contains 30 species already present in at least one of the four countries of the focus area, and 42 species not yet present. This approach allowed investigating the factors affecting the spread and distribution of the worst current as well as future biological invasions across the focus area.

#### Species occurrence data

Information on the current global (i.e. native and invaded) spatial distribution of the 72 species was obtained from the following international and regional data gateways: Global Biodiversity Information Facility (GBIF, http://data.gbif.org), Biological Collection Access Service for Europe (BioCase, http://www.biocase.org), Ocean Biogeography Information System (IOBIS, http//:iobis.org/mapper), Netherlands Biodiversity Information Facility (NLBIF, http://www.nlbif.nl), Waarnemingen network (http://waarnemingen.nl), National Biodiversity Network (NBN, Gateway http://data.nbn.org.uk), Discover Life (http://www.discoverlife.org) and an extensive ISI Web of Knowledge literature review (S3 Table).

Once we obtained a satisfactory global distribution map for a species, the software ENMTools v1.3 (enmtools.blogspot.co.uk/ [22]) was used to remove duplicate records using one of the environmental grids (see below) as reference. This procedure leaves only one occurrence point per pixel of 5 arcminutes (approximately 9×9 km at the equator), thereby reducing the spatial bias (i.e. clustering) of occurrence data and substantially improving the reliability of predictions [23].

#### Continental variables

Due to the high number of species modelled, we chose a relatively high resolution of 5 arcminutes for variables used as predictors in SDM. This resolution was the best compromise between reducing computation space and time without notably loosing predictive performance. All layers were used with a global coverage and WGS84 reference coordinate system.

To model the potential distribution of terrestrial and freshwater organisms, eight global bioclimatic variables plus geographic elevation were obtained from WorldClim (http://www.worldclim.org): (1) annual mean temperature (°C), (2) temperature seasonality (standard deviation) (°C), (3) maximum temperature of the warmest month (°C), (5) minimum temperature of the coldest month (°C), (6) annual precipitation (mm), (7) precipitation of the driest month (mm), (8) precipitation seasonality (coefficient of variation) (mm), and (9) altitude (m). These bioclimatic variables represent annual trends, seasonality and extremes for species survival and were thus selected based on their meaningfulness to explain the large scale distribution of species. Indeed, bioclimatic variables have been commonly used to calibrate distribution models, both for terrestrial (e.g. [24, 25]) and freshwater (e.g. [26, 27]) invasive species. In spite of certain inter-correlation between variables (Pearson correlation *r* < |0.8|, as checked with ENMTools v1.3, S4 Table), the modelling algorithm used (see Model calibration and projection) has inbuilt methods for regularization and is more stable to correlated variables than other classic regression techniques, so according to Elith *et al*. [28] there is less need to remove correlated variables.

Although previous studies suggest that the influence of geological makeup on the distribution of invasive species is rather limited (e.g. [26]), we included a geological variable as a proxy for water, sediment and soil characteristics (such as nutrient concentration and pH), which are potentially relevant to invasive species. Data on global onshore geological units were obtained from the Commission for the Geological Map of the world (CCGM-CGMW, Paris 2010, http://ccgm.free.fr/) and included seven bedrock geologies: endogenous plutonic or metamorphic rocks, extrusive volcanic rocks, island, lake, ophiolitic complex, sedimentary rocks and undifferentiated facies.

In addition to environmental factors, we introduced five anthropogenic variables as proxies of the human footprint: the Global Human Influence Index (HII, a measure of human perturbation), Land cover, Population density, Port proximity and Road proximity. (1) The Global Human Influence Index map (Socio-Economic Data and Applications Centre, http://sedac.ciesin.columbia.edu) consists of a composite of global data layers on factors presumed to exert an influence on ecosystems: human population distribution, urban areas, roads, navigable rivers, and various agricultural land uses. The sums of degradation scores for each of these factors constitute the HII layer, which ranges from 0 (close to pristine locations), to 64 (much degraded areas) [18]. (2) Data on global Land cover was obtained from IGBP- International Geosphere-Biosphere Programme (MODIS Global Land Cover Classification v2, http://www-modis.bu.edu/landcover) and included nine categories: forest, shrubland, savannah, grassland, wetland, cropland/natural vegetation, urban, snow/ice and barren/sparsely vegetated. (3) The density of human Population has been shown to affect the broad scale distribution of invasive species (e.g. [10, 29]), and it was thus included as a separate layer (Oak Ridge National Laboratory, http://www.ornl.gov/sci/landscan). Finally, Port and Road proximity were calculated using the euclidean distance (in km) to (4) the closest commercial port (defined as those with >30 megatonnes total cargo volume in 2009) and (5) the closest primary road calculated in ArcView v10.0 from basic ESRI global maps as described in Gallardo & Aldridge [20]. Despite the inclusion of HII, which may partially account for some of the other four human footprint proxies selected, collinearity levels were low (Pearson *r*<|0.6|, S5 Table).

Positive correlation between biodiversity and human population has been detected for several groups and geographic regions, and this relationship has been attributed, amongst other factors, to uneven sampling effort [30]. Sampling bias towards highly populated areas can compromise the results of our models, since a significant role of the human footprint in shaping invasive species distribution could be an artefact explained by organisms being most frequently sampled and reported from densely populated areas. To investigate the issue, a sampling effort map was generated by creating a density map for the “target group”, i.e. the 72 species investigated [31]. This density map reflects spatial differences in sampling effort in, for instance, highly populated areas of Europe, North America and Australia (S1 Fig). This sampling effort map was nevertheless not significantly correlated to human footprint proxies (Pearson *r*<|0.2|, S5 Table). Whilst the lack of direct correlation does not necessarily imply that species maps are free from bias, we conclude that uneven sampling effort is unlikely to compromise the results from our models.

#### Oceanic variables

For modelling of marine species, a range of geophysical, biotic and climatic data was downloaded from Bio-Oracle (Ocean Rasters for Analysis of Climate and Environment, http://www.oracle.ugent.be) at a spatial resolution of 5 arcminutes [32]. After checking the correlation of layers with ENMTools v1.3, the following 12 variables (with Pearson’s *r*<|0.8|, S6 Table) were selected based on their potential relevance to explain the distribution of marine invasive species: (1) maximum surface temperature (°C), (2) minimum surface temperature (°C), (3) maximum Photosynthetic Active Radiation (PAR, Einstein/m^2^/day), (4) salinity (PSS), (5) pH, (6) phosphate (μmol/L), (7) nitrate (μmol/L), (8) dissolved oxygen (ml/L), (9) calcite (mol/m^3^), (10) silica (μmol/L), (11) minimum chlorophyll-*a* (mg/m^3^), and (12) maximum chlorophyll-*a* (mg/m^3^).

In addition, we included a map of Marine Human Impacts (MHI [33]). Similar to the Human Influence Index, this map summarises information on 17 human activities that directly or indirectly impact marine ecosystems. These include fishing, shipping, pollution, location of benthic structures and population pressure (more information at http://www.nceas.ucsb.edu/globalmarine/impacts). In addition, minimum and maximum chlorophyll-*a* can be used as indicator of eutrophication resulting from coastal activities (i.e. agriculture, aquaculture, sewage) [34], and was therefore used as an indirect proxy for the human influence on marine habitats. For the purpose of this study, we considered the upwelling of nutrient-rich water in certain areas of the world unlikely to affect the distribution of marine invasive species, yet this source of chlorophyll-*a* cannot be disregarded.

#### Model calibration and projection

To investigate the relative importance of environmental factors and the human footprint in determining the global distribution of invasive species, we used the Maxent algorithm, which typically outperforms other methods based on predictive accuracy [35, 36]. As defined by Phillips *et al*. [37], Maxent is a machine learning algorithm that estimates the species probability distribution of maximum entropy subject to the set of constraints that represent our (incomplete) information about the species distribution and the environmental factors that might limit it. For input, Maxent models use the dataset of species occurrences and the set of environmental and human footprint predictors that might affect the likelihood of species establishment. To calibrate models, modelling parameters described by Gallardo & Aldridge (2013a) were implemented in software MaxEnt v3.3k (www.cs.princeton.edu/~schapire/maxent): convergence threshold = 105, maximum iterations = 500, prevalence = 0.5, occurrence data split into 70% of the data for modelling and 30% for testing; 10,000 random background points generated from the global-wide background. As recommended by Merow *et al*. [36], background choice was selected on the base of the study goal (identifying general macroecological patterns of distribution) and the type of species modelled (globally invasive species).

The ‘regularization multiplier’ parameter affects how focused or closely-fitted the output predicted distribution is [28]. For instance, a larger value than the default of 1 will result in a more spread out, less localized prediction (Phillips & Dudík, 2008), which might be useful when modelling invasive species. Following Gallardo *et al*. [26], a regularization multiplier of 1 to 4 was tested in this study and models compared using ENMTools [22]. The Akaike information criterion corrected for sample size (AICc) was used to select the best regularization option, as recommended by Warren & Seifert [38].

Once the optimum regularization was defined, we tested the inclusion of variables in the model. Following the approach for model selection in MaxEnt proposed by Warren *et al*. [38], we sequentially removed one variable at a time and selected the model with lowest AIC_c_, using a threshold of ΔAIC = 5. This stepwise removal was done manually, since automatic variable selection is not yet implemented in MaxEnt’s console. The procedure removes variables that are redundant or not relevant in explaining the species’ distribution, and has previously been used to optimize SDM calibration [26, 39]. Unfortunately, computer restrictions limited the use of more complex options for model selection, such as selecting a number of equally plausible models based on different combination of predictors [40]. Because comparing methods for variable selection in SDM is out of the scope of this study, and stepwise selection based on AIC has been shown to perform similarly to other more exhaustive algorithms [41], we believe this method provides the best option for the purpose of this study.

Among the different measures of variable importance provided by MaxEnt, we chose permutation importance, which is calculated by randomly permuting training presence and pseudo-absence data. In the absence of adequate variance partitioning methods for MaxEnt, permutation importance values were used as gross estimates of the variance explained by variables or groups of variables in SDM. It has to be noted, however, that permutation importance values are not strictly additive and do not account for shared variance between variables.

To investigate the response of invasive species to the most important predictors identified through SDM, suitability scores calculated by MaxEnt for each habitat-taxon group (i.e. terrestrial plants, terrestrial animals, freshwater and marine organisms) were plotted against each of the most important predictors, and a regression curve was added using univariate Generalized Additive Models (GAM [42]) in R v.3.0 [43]. GAM was chosen instead of other regression procedures because of its ability to deal with nonlinear relationships between the response and the predictor (Guisan et al. 2002).

To assess model performance, we used two metrics of model fit: the area under the ROC curve (AUC) (Hanley & McNeil 1982), and the sensitivity of the model. The AUC represents the probability that a random occurrence locality will be classified as more suitable than a random background point, and is one of the most popular metrics of fit in the MaxEnt literature [36]. However, AUC has been criticized because it ignores continuous suitability scores, and tends to offer higher values when increasing the geographical extent of the study and the number of background points [44]. In addition, AUC weights commission (false absence) and omission (false presence) errors equally, while they might not have the same importance depending on the study’s goal [44]. For the purpose of investigating the potential distribution of invasive species, omission errors represent a more serious drawback of SDM, since apparent absences (commission errors) may be due to low detectability of the species, and the species might be able to invade in the future. For this reason, we used the percentage of real occurrences correctly classified by the model (sensitivity) as a supplementary measure of fit. While both metrics have their limitations (see for instance [45]), they offer complementary information that can be used to assess the overall fit of SDM.

After calibration, models were projected to obtain suitability maps. Suitability is a measure of the match with the environmental and human conditions of locations currently invaded by a species and ranges from 0 (completely dissimilar) to 1 (perfect match). The threshold minimizing omission and commission errors of the model was used to transform suitability maps into a predicted presence/absence map [46]. Thresholded maps provided a simple prediction for each species allowing identifying broad geographic regions where the human footprint together with suitable environmental conditions may facilitate the successful establishment of an invasive species.

Finally, all maps were combined together into a cumulative global ‘heat map’ representing the total number of species predicted present. Subsequently, we focused on the focus area (i.e. Great Britain, France, Belgium and The Netherlands) for closer examination, which allowed locating potential hotspots for species invasion that would be difficult to discern using the global projection.

## Results

### Major predictors of invasive species’ global distribution

**Fig 1:**
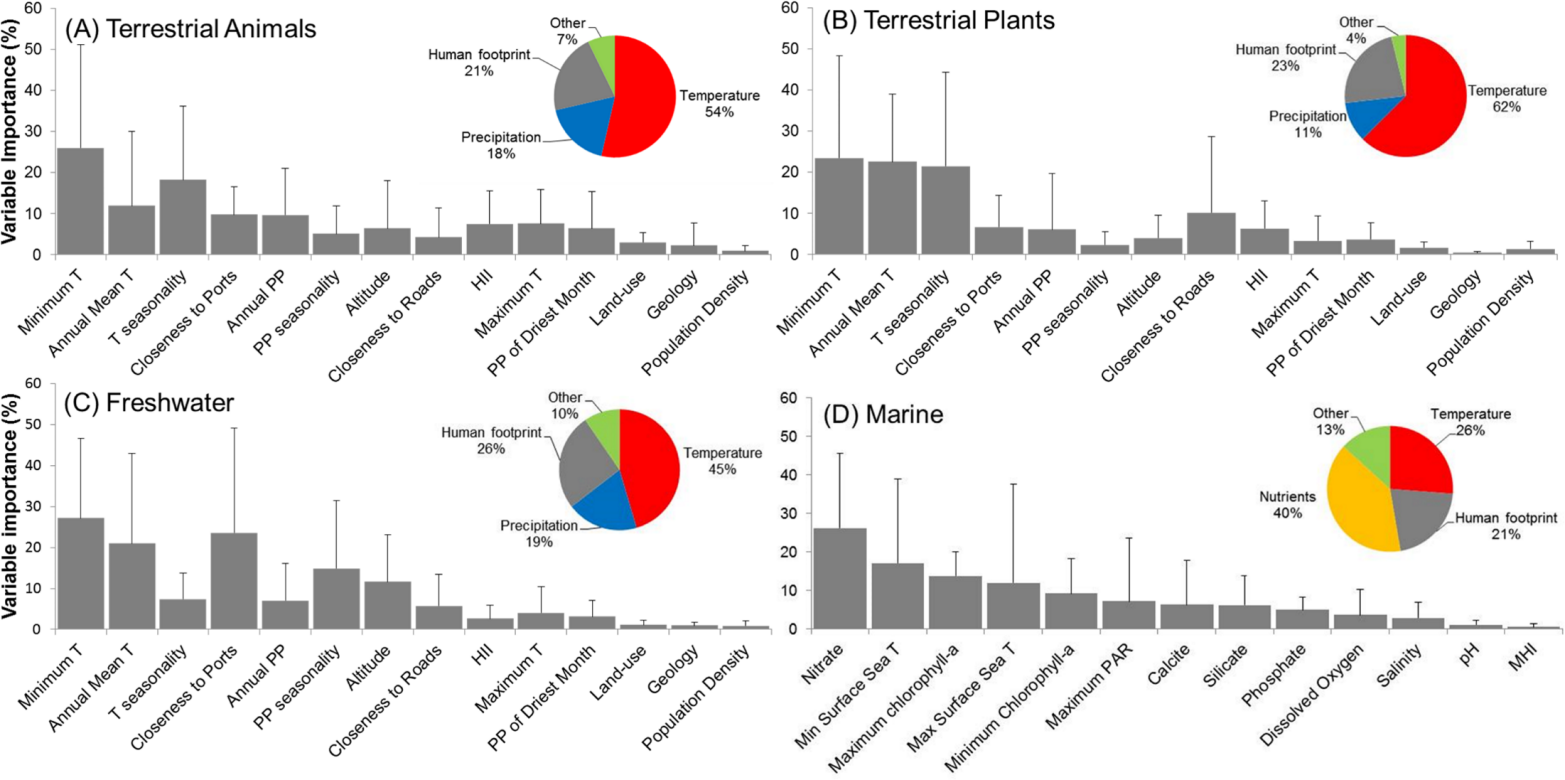
Permutation importance of environmental and socio-economic predictors in species distribution models. Variables are ordered by their overall mean importance. T = temperature, PP = precipitation, HII = Human Influence Index, MHI = Marine Human Influence. Bars represent the standard deviation of the mean value. Insert pie-charts summarize the influence of major groups of variables on the distribution of the four taxon-habitat groups. Temperature related variables were most important in explaining invasive species distribution, followed by the human footprint.

**Fig 2:**
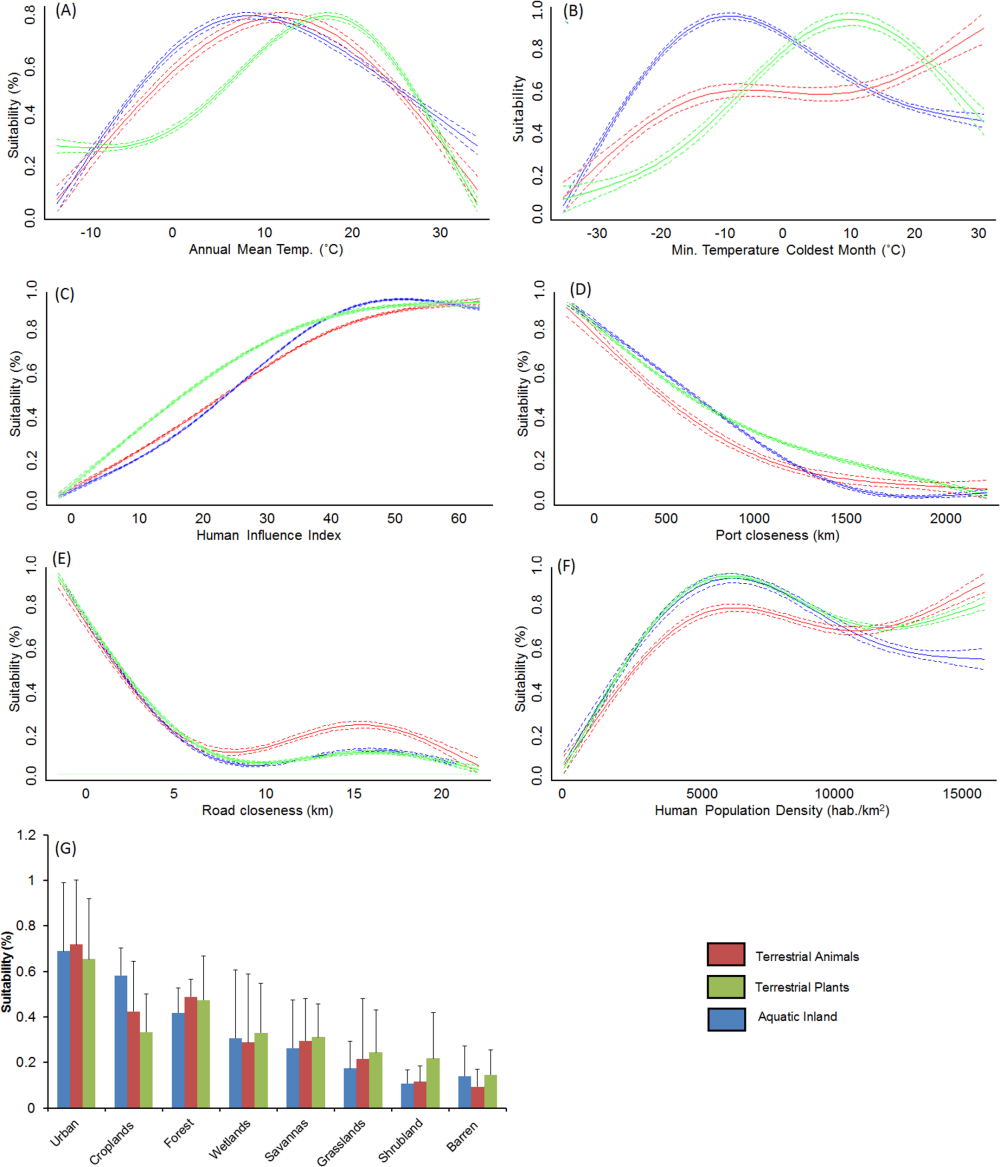
Response curves showing the relationship between spacial suitability scores extracted with MaxEnt and seven of the most important drivers of their global distribution. Lines represent the combined response of terrestrial animals (red), terrestrial plants (green) and freshwater organisms (blue) evaluated in this study. Pointed lines represent 95% confidence intervals around the mean. Spatial suitability for invasive species generally showed a unimodal response to temperature related variables, increased with Human Influence and population density, and closeness to transport networks (roads and ports).

**Fig 3:**
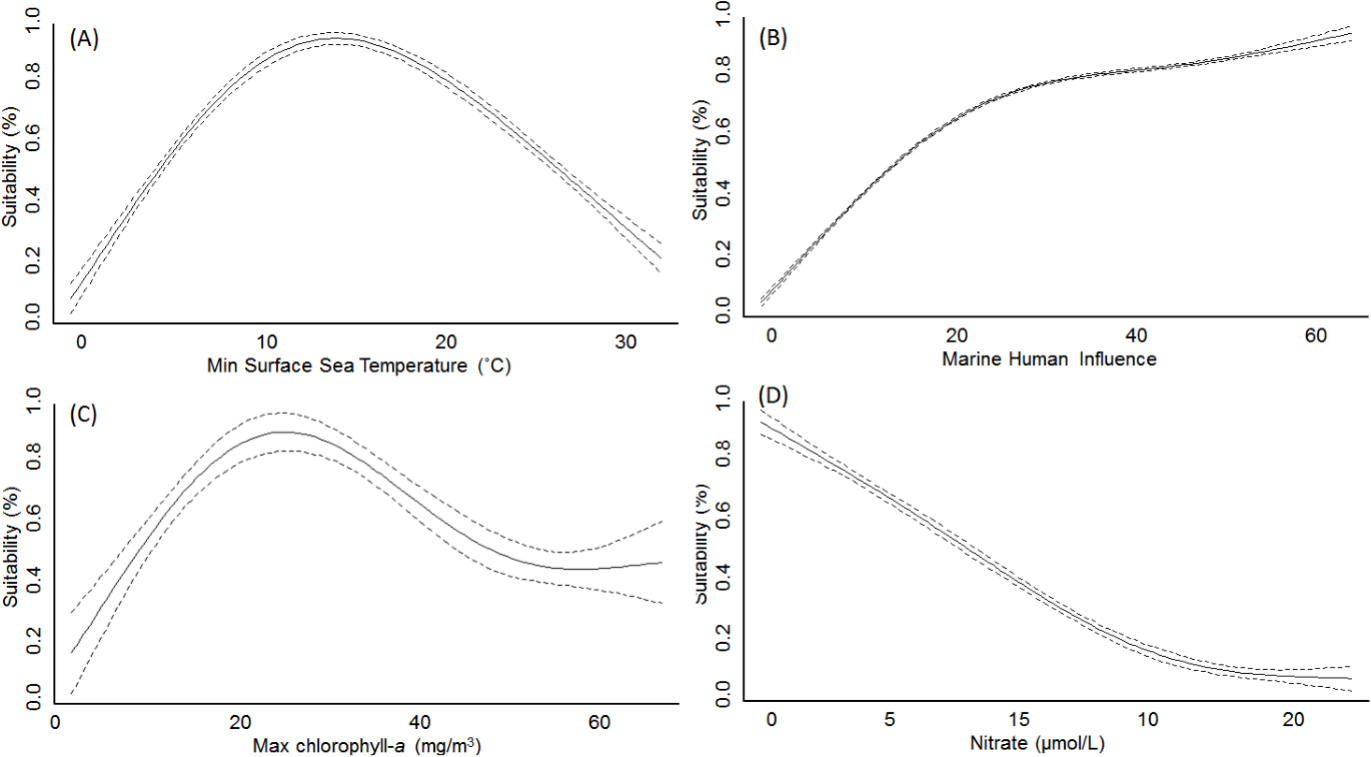
Change in spatial suitability for marine organisms along the most important drivers of their global distribution. Pointed lines represent 95% confidence intervals around the mean. Like in Fig 2, the spatial suitability for marine invaders was highest at intermediate temperatures and increased with Human Influence.

The AUC of models ranged between 0.90 and 0.99 (average 0.97±0.02), and sensitivity between 0.73 and 1.0 (average 0.91±0.05), thereby suggesting a relatively high performance. The analysis of model outputs grouped by four major habitat-taxon groups revealed certain generalities in the response of terrestrial plant and animal, freshwater and marine invasive species to global environmental and human gradients (see extended results from SDM in S7 and S8 Tables). As expected, temperature related variables explained the largest amount of the potential distribution of the invasive species investigated (average for terrestrial animals = 53.6%, terrestrial plants = 62.5%, freshwater = 45.4%, marine = 26.2%; Fig 1). Aquatic inland organisms, terrestrial plants and terrestrial animals showed a similar response to mean annual temperature (Fig 2A), peaking around 10°C; but different suitability optima at increasing minimum temperatures (Fig 2B). The response of marine invaders to minimum water temperature was variable although it generally peaked at 15°C (Fig 3A).

The permutation importance of the human footprint was highly variable, with values between 1.2 and 87.3% (average for terrestrial animals = 21.4%, terrestrial plants = 23.0%, freshwater = 25.8%, marine = 21.1%, Fig 1). Proximity to ports, roads, the Human Influence Index (HII) and cholorphyll-*a*—used as proxy for oceanic eutrophication—were particularly important factors (Fig 1). Invasive species showed a positive response to increasing human influence values, with HII>25 leading to habitat suitability scores over 50% for most species (Fig 2C). This factor reached highest permutation importance in models developed for terrestrial animals, particularly for insects such as the Mediterranean fruit-fly (*Ceratitis capitata*) and the Argentine ant (*Linepithema humile*). In the marine environment, most species also showed increasing occurrence potential with cumulative Marine Human Impact (MHI) values (Fig 3B), although this variable achieved lower permutation importance than expected (<1%). Suitability for marine invaders peaked at chlorophyll-*a* concentrations around 20–30 mg/m^3^ (Fig 3C). An important driver of the distribution of freshwater invaders was proximity to commercial ports (Fig 1C and Fig 2D), which achieved >40% permutation importance in the models calibrated for the walking catfish (*Clarias batrachus*) and American shrimp (*Gammarus fasciatus*) (S3 Table). Proximity to roads was a particularly important driver for plant species distribution. In fact, the distribution of two plants, the Kudzu (*Pueraria lobata montana*) and Kahili ginger (*Hedychium gardnerianum*) was largely explained by road proximity (permutation importance>30%), showing the maximum probability of invasion within 2 km from roads (as in Fig 2E). Population density and land-use were the least significant predictors of invasive species distribution included in the models. Nevertheless, population density>2500 habitants/km^2^ led to invasion suitability >50% (Fig 2F), whereas urban and agricultural areas were the land-uses under highest risk of invasion (Fig 2G).

### Mapping invasive species distribution at global and regional scale

**Fig 4:**
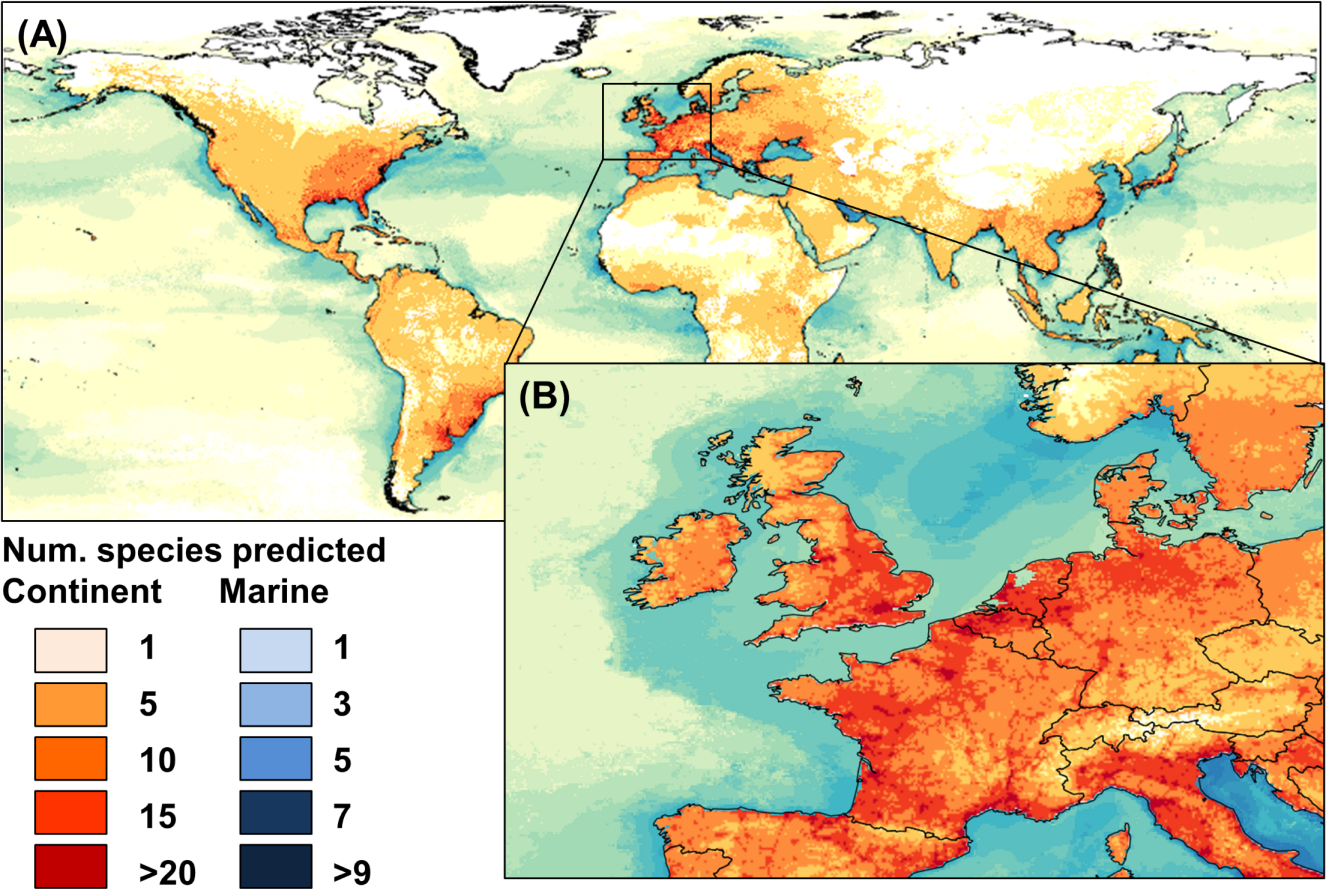
Heat map showing cumulative probability of presence of 72 invasive species at (A) the global scale, and (B) across the focus area in Great Britain, France, Belgium and The Netherlands. High cumulative risk scores can be found around ports and urban areas around the British Channel and southern part of the North Sea.

The ‘heat map’ presented in Fig 4 synthesizes information regarding the risk associated with a diverse set of aquatic and terrestrial organisms displaying a wide range of origins, pathways of invasion and habitat preferences. In the focus area (Fig 4B), the ‘heat map’ highlighted ports and urban areas around the British Channel and southern part of the North Sea as potential hotspots of invasive species. Occurrence potential decreased towards the NW in Great Britain, and towards the SE in France, Belgium and The Netherlands. The surroundings of commercial ports like the Thames, Southampton, Rotterdam, Antwerp and Boulogne-Sur-Mer showed the highest number of potential future invaders, with up to 36 different invasive species showing values above the selected likelihood threshold. Major urban areas like London, Liverpool, Paris, Amsterdam, Utrecht, Ghent or Brussels can also be important gateways of invasion according to our future ‘heat map’. In the marine environment, the combined suitability for invaders was higher towards the north-east of the North Sea, along the coast of Norway (Fig 4B).

## Discussion

### Major predictors of invasive species’ global distribution

Confirming observations made in previous studies that implemented climatic and non-climatic factors [mostly land cover, e.g. 29; but see 20 for other human activities], climate-related variables in our models were the most important in determining the global scale distribution of 65 out of 72 invasive species analysed. Amongst climate factors, temperature-related variables explained about half of the distribution of terrestrial and freshwater organisms, which is in accordance with previous studies [20, 27, 47].

Araujo *et al*. [48] revealed that differences in the global distribution of species are largely driven by their cold tolerance level, with upper temperature tolerance levels being relatively similar across species. Likewise, our models showed that maximum temperature optima are centred around 25°C for all continental organisms, and that optimum minimum temperatures are generally lowest for freshwater organisms, medium for terrestrial plants, and highest for terrestrial animals. Water **t**emperature was also a primary driver of biological invasions in the marine environment, with species spanning a wide range of suitable water minimum temperatures, from 5 to 25°C. This partially confirms the observation of Zerebecki & Sorte [49] that marine invaders tend to occupy habitats with broader temperature ranges and higher maximum temperatures than natives.

Despite the dominant role of temperature in SDM, the human footprint explained about a quarter of the global distribution of invasive species, with only minor differences between terrestrial and aquatic habitats (Fig 1). Although results were very variable between species, models revealed common patterns across the four habitat-taxon groups investigated. Firstly, all groups—but particularly invasive plants—featured distributions that were influenced by the location of transport routes. This confirms previous authors who argued that transport networks promote the dispersal of non-native species by altering habitats, stressing native species, and providing movement corridors [12, 16, 19, 50]. For instance, roads have been shown to promote hunting, fishing, passive harassment of animals, and landscape modifications [50]. Transport routes therefore enhance immigration rates of new species and the spread of non-native species that have already arrived.

All of the modelled species showed a consistent positive response to the Human Influence Index and the Marine Human Impacts, which suggests that the probability of an invasion grows with the intensity of habitat use by humans [20, 51]. For a varied range of organisms, the probability of invasion exceeded 50% at Human Influence values above 25, which at the global scale correspond to the most industrialized regions of Europe, North America, South East Asia and South America (S2 Fig). Moreover, regions with Human Influence values above 25 largely coincide with global hotspots of invasive species richness recently reported by Leprieur *et al*. [51] and Béllard *et al*. [29]. This is presumably because the human activities responsible for the introduction of non-native species such as horticulture, pet trade, hunting or fishing, are more common in densely populated areas. Amongst species modelled in this study, the distribution of insects was particularly strongly affected by the Human Influence Index. Although invasive insects can fly over short distances and are also carried by the wind and animals, their main long-distance dispersal is human-assisted, as contaminants of imported plants, soil, flowers or fruit [52]. Despite the positive response of marine invaders to the degree of human influence, Marine Human Impacts registered low permutation importance, even though this factor summarized the effect of multiple human activities that have direct or indirect impacts on marine ecosystems (e.g. shipping, fishing, pollution). In contrast, primary productivity (chlorophyll-*a*)—used in this study as a proxy for eutrophication although it can also indicate water upwelling—was an important factor in explaining the distribution of marine invasions. These observations, together with the high permutation importance showed by temperature and nutrients such as nitrate, support a relevant role of the match between physicochemical characteristics of the donor and receiving waters for marine invasions, as suggested by Seebens *et al*. [53].

Proximity to ports was identified as an important predictor not only for aquatic species that can be transported in ballast water or attached to the ship’s hull, but also for some terrestrial plants and animals. The importance of ports as gateways for non-native species is well known, with shipping intensity and distance between origin and recipient ports having been identified as the most important factors determining invasion risk [53]. Invasive species are transported as commodities and deliberately released or escape from captivity, or can be involuntarily transported as contaminants or stowaways [12, 54]. A further source for the importance of port proximity may lay in the fact that port closeness also reflects coastline proximity. Coastal landscapes are being transformed as a consequence of the increasing demand for infrastructures to sustain residential, commercial and tourist activities. Thus, intertidal and shallow marine habitats are largely being replaced by a variety of artificial substrata (e.g. breakwaters, seawalls, jetties) that are very susceptible to invasion [55]. In addition, invasive species are usually tolerant to harsh environmental conditions and may benefit from reduced native biodiversity—thus biotic resistance—in inter-tidal areas. Finally, population density and land-use were often removed from models during variable selection in favour of other socio-economic indicators, which suggest that their effects were already accounted for by factors such as the Human Influence Index, road and port proximity.

### Mapping invasive species distribution at regional scale

Spatial patterns of invasion in the focus area suggest there is room for expansion of current as well as future invaders, with vast areas highly suitable for up to 36 different species, which poses a tremendous challenge in terms of prevention and management. Spatially, the SE of England and coastal areas of Belgium and The Netherlands are under the highest risk of multiple invasions, with risk progressively decreasing outwards, i.e. north and westwards in Great Britain, and south and eastwards on the continent. We can therefore consider the north-eastern part of the focus area (and in particular urban areas adjacent to major ports like London, Oostende, Zeebrudge, Rotterdam and Antwerp) as a hot spot of invasion. The inclusion of human footprint proxies into SDMs was translated into highest cumulative risk scores in close relation to the location of commercial ports, transport networks, population density and the intense use of landscapes for industry, urbanization or recreation purposes (i.e. high propagule pressure), which altogether amplifies the potential for invasion.

In the marine environment, the suitability for invaders was highest around the ports of the British Channel and southern part of the North Sea. That said, in general the focus area was predicted as fairly unsuitable for most of the potential (not yet present) marine species. This is likely due to the fact that these species mostly originate from the Pacific Ocean and Mediterranean Sea, both of which are characterized by rather different climatic conditions from the focus area. This observation agrees with a recent study about the risk of marine invasions caused by global shipping [53]. In spite of their substantial maritime traffic volume, no European port was included in the rank of the 20 ports with highest invasion risk, a fact that authors attributed to the lack of match in environmental conditions between donor and host ports. This supports our prediction that the risk of new marine invasions in the focus area is relatively low, at least from some of the world’s worst invaders investigated here.

We are aware that the analyses performed in this study suffer from multiple limitations. First, results from this study refer to the species investigated and further studies with a more representative set of invaders are needed to generalize our interpretations. In terms of predictors, although the factors evaluated in this study provide valuable information on the effect of the human footprint on invasive species, the inclusion of other predictors more directly related to propagule pressure and species dispersal (e.g. aquaculture, horticulture, shipping frequency) may further improve the predictive capacity of models. In addition, variables related to habitat conditions at regional to local scale, such as vegetation state, water and soil chemistry or habitat structure, might be helpful in refining the areas under greatest threat. In terms of modelling, a number of factors may affect the accuracy of SDMs, some of which are related to the unrealistic ecological assumptions behind models (e.g. species in equilibrium with environment, no dispersal limitations, lack of biotic interaction) and extensively described elsewhere (e.g. [35, 37]). Sample size, bias and clustering of species’ occurrences may affect the modelled distribution of a species [23], and were thus treated with careful consideration. Bias towards highly accessible areas due to uneven sampling effort is a common and pervasive problem in species occurrence data, and may partly account for the importance of human footprint variables in our models. The extent to which sampling bias and propagule pressure has contributed to the patterns described in this study is difficult if not impossible to discern. We therefore advocate a cautious evaluation of data quality and testing different modelling options to optimize final predictions.

Overall, our study provides a comprehensive overview of the relative importance of environmental factors and the human footprint to explain the global scale occurrence of invasive species. Analyses suggest that the occurrence of some of Europe’s most damaging invaders is primarily limited by temperature tolerance. Nonetheless, the human footprint explained on average a quarter of invasive species distribution and was directly linked to the vectors and pathways of distribution for invasive species. Amongst human related factors, transport networks were the most influential over the distribution of invasive plants; terrestrial animals were favoured by the degradation of natural ecosystems; port proximity determined the presence of freshwater invaders; and eutrophication was closely related to marine invasions. These findings are of primary importance since they confirm that the relationship between invasive species and the human footprint–widely investigated at local to regional levels—can be scaled up to the global level. Spatial predictions further confirmed the capacity of the human footprint to correct suitability scores by promoting highest risk values in areas where propagule pressure can be presumed high (i.e. close to transport networks and densely populated areas). These results, consistent across a wide range of species and habitats, suggest that climate is important, but not enough to anticipate future invasions. Consequently, human related information—currently available in the form of easily accessible maps and databases—should be routinely implemented into predictive frameworks to inform upon geographically targeted policies to prevent and manage invasions. Otherwise we might be seriously underestimating the species and areas with the highest risk of invasion. Yet, current linkages between SDM modelling and species management are currently weak and require further progress [56].

## Supporting Information

S1 Table List of data sources consulted to select the ‘worst’ invasive species in the focus area across Great Britain, France, Belgium and The Netherlands. (PDF)

S2 Table Invasive species selected for modelling. (PDF)

S3 Table References used to complete the known native and invasive distribution of species. (PDF)

S4 Table Correlation between continental layers used for calibrating Species Distribution Models. (PDF)

S5 Table Correlation between human footprint variables and the sampling effort map. (PDF)

S6 Table Correlation between marine layers used for calibrating Species Distribution Models. (PDF)

S7 Table Species Distribution Model (SDM) output statistics for terrestrial plants, terrestrial animals, and aquatic inland species. (PDF)

S8 Table Species Distribution Model (SDM) output statistics for marine invaders. (PDF)

S1 Fig Sampling effort map generated to investigate the species-people correlation. (PDF)

S2 Fig Map of global Influence Index (HII). (PDF)
