# Supplementary material for: The Importance of the Human Footprint in Shaping the Global Distribution of Terrestrial, Freshwater and Marine Invaders
Source: PLoS One. 2015 May 27;10(5):e0125801. doi: 10.1371/journal.pone.0125801 (PMC4446263; doi:10.1371/journal.pone.0125801)
Supplement: S1 Table — (PDF) [file pone.0125801.s001.pdf]

Table S1. List of data sources consulted to select the 'worst' invasive species in the focus area across Great Britain, France, Belgium and The Netherlands. #Num: number of species included in the 'metalist' from each provider. Some species were listed in more than one of the consulted lists.

Provider	Details	Geographic scope	#Num	Source
DAISIE (Delivering Alien Invasive Species Inventories for Europe)	100 of the worst European invasive species	Europe, Israel and part of Russia	100	http://www.europe-aliens.org
IUCN's Invasive Species Special Group (ISSG)	100 of the world's worst invasive species. Species native to Europe were not considered.	Global	83	http://www.issg.org
Black List of Europe	Crop pest were not considered	Europe	211	http://wcd.coe.int
ICES Working group on introduction and transfer of marine organisms (WGITMO)	Marine invasive species	Global	7	http://www.ices.dk/ workinggroups/
SEBI2010 (Streamlining European 2010 Biodiversity Indicators)	'Trends in invasive alien species' indicator	Europe	56	http://www.bipnational.net/IndicatorInitiatives/ SEBI2010
EPPO (European and Mediterranean Plant Protection Organization) List of invasive alien plants	EPPO list of invasive plants	Europe, Mediterranean region, eastern Europe and Russia	34	http://www.eppo.int/ INVASIVE_PLANTS/ias_lists.htm#IAPList
BFIS-Harmonia (Belgian Forum on Invasive Species)		Belgium	52	http://ias.biodiversity.be/
Waarnemingen network		Belgium and The Netherlands	9	http://waarneming.nl http://waarneming.be

NOBANIS (North European and Baltic Network on Invasive Alien Species)		Northern Europe	82	http://www.nobanis.org/Factsheets.asp
Panov *et al.* (2009)	Aquatic inland invasive species	Europe	24	http://www.reabic.net/publ/IEAM2009_Panov_etal.pdf
Nentwig *et al.* (2010) Kumschick & Nentwig (2010)	Terrestrial animals	Europe	7	http://www.cdt.ch/files/docs/1be5b9948981b5baead5de5936237e0f.pdf<br><br>http://ftpshare.its.unibe.ch/iee/pub/7/2010/960.pdf
Gallardo & Aldridge (2013a, b)	Aquatic inland invasive species	Great Britain and Ireland	21	http://www.esajournals.org/doi/abs/10.1890/12-1018.1
