# Supplementary material for: The Importance of the Human Footprint in Shaping the Global Distribution of Terrestrial, Freshwater and Marine Invaders
Source: PLoS One. 2015 May 27;10(5):e0125801. doi: 10.1371/journal.pone.0125801 (PMC4446263; doi:10.1371/journal.pone.0125801)
Supplement: S2 Table — (PDF) [file pone.0125801.s002.pdf]

Table S2. Invasive species selected for modelling. Total= 72 species. Native Continent: As= Asia, SAm= South America, Eur= Europe, Nam= North America, Af= Africa, Au=Australia, Unknown= native origin not known. Invaded country: countries in the focus area where the species has been already reported. GB: Great Britain, FR: France, BE: Belgium and NTL: The Netherlands. Checklists: lists of “worst” potential invaders in the study area in which the species was included. EU-Black: Black List of invasive alien species entering Europe; DAISIE: Delivering Alien Invasive Species Inventories for Europe; SEBI: Streamlining European 2010 Biodiversity Indicators; NOBANIS: North European and Baltic Network on Invasive Alien Species; GBNNSS: Great Britain’s Non-Native Species Secretariat; Harmonia: Belgian Forum on Invasive Species; Waarneming: Alarm list of invasive species in The Netherlands; WGITMO: ICES Working group on introduction and transfer of marine organisms; ISSG: IUCN’s Invasive Species Special Group; G&A: Gallardo and Aldridge (2013a, b); Panov: Panov et al. 2009; EPPO: List of invasive alien plants. Check the geographic scope of each consulted list and data sources in Table 1.

**Phylum**	**Species name**	**English name**	**Native Continent**	**Invaded countries**	**Checklists**
Algae	*Asparagopsis taxiformis*	Limu kohu	Nam	0	EU-Black
Algae	*Caulerpa taxifolia*	Caulerpa	As, Nam	GB, FR, NTL	EU-Black, DAISIE, SEBI, ISSG
Algae	*Codium fragile*	Green sea fingers	As	GB, BE, NTL	DAISIE
Algae	*Coscinodiscus wailesii*		As, Nam	GB, FR, BE, NTL	EU-Black, DAISIE, SEBI, GBNNSS
Algae	*Undaria pinnatifida*	Japanese kelp	As	GB, FR, BE, NTL	EU-Black, DAISIE, SEBI, WGITMO, ISSG
Angiospermae	*Acacia dealbata*	Silver wattle	Au	FR	EU-Black, DAISIE, EPPO
Angiospermae	*Cabomba caroliniana*	Carolina Water-shield	SAm	GB, FR, BE, NTL	EU-Black, Waarneming, EPPO
Angiospermae	*Carpobrotus edulis*	Hottentot-Fig	Af	GB, FR, BE	EU-Black, DAISIE, SEBI, GBNNSS, Harmonia, Waarneming, EPPO
Angiospermae	*Cortaderia selloana*	Pampas Grass	SAm	GB, FR, BE	EU-Black, DAISIE, SEBI, Waarneming, EPPO
Angiospermae	*Echinocystis lobata*	Wild Cucumber	NAm	FR	EU-Black, DAISIE, SEBI, Waarneming
Angiospermae	*Eichhornia crassipes*	Water Hyacinth	SAm	GB, FR, BE, NTL	ISSG
Angiospermae	*Hedychium gardnerianum*	Kahili ginger	As	GR	EU-Black, DAISIE, SEBI, ISSG
Angiospermae	*Imperata cylindrica*	Blady grass	As	0	ISSG
Angiospermae	*Lantana camara*	Ach man	SAm	0	ISSG

Angiospermae	*Melaleuca quinquenervia*	Melaleuca	Au	0	ISSG
Angiospermae	*Miconia calvescens*	Bush currant	SAm	0	ISSG
Angiospermae	*Mikania micrantha*	American rope	SAm	0	ISSG
Angiospermae	*Myriophyllum heterophyllum*	Twoleaf Watermilfoil	Unknown	FR, BE. NTL	EU-Black, Harmonia
Angiospermae	*Opuntia ficus-indica*	Prickly-pear cacti	SAm	FR	EU-Black, DAISIE, SEBI
Angiospermae	*Oxalis pes-caprae*	Bermuda-buttercup	Af	GB, FR	EU-Black, DAISIE, SEBI, GBNNSS, EPPO
Angiospermae	*Prosopis glandulosa*	Honey mesquite	NAm	0	ISSG
Angiospermae	*Pueraria lobata montana*	Kudzu	As	0	EU-Black, ISSG
Angiospermae	*Rubus ellipticus*	Asian wild raspberry	As	0	ISSG
Angiospermae	*Schinus terebinthifolius*	Brazilian holly	SAm	0	ISSG
Angiospermae	*Tamarix ramosissima*	Salt cedar	As	0	ISSG
Annelida	*Marenzelleria neglecta*	Red-gilled Mud Worm	NAm	BE. NTL	EU-Black, DAISIE, SEBI, NOBANIS, WGITMO
Arthropoda	*Aedes albopictus*	Asian Tiger Mosquito	As	FR, BE, NTL	EU-Black, SEBI, Waarneming, ISSG
Arthropoda	*Anoplolepis gracilipes*	Yellow crazy ant	Af	0	ISSG
Arthropoda	*Anoplophora chinensis*	Citrus longhorn beetle	As	BE, NTL	EU-Black, DAISIE, SEBI, GBNNSS, Waarneming
Arthropoda	*Anoplophora glabripennis*	Asian longhorn beetle	As	GB, FR	EU-Black, DAISIE, SEBI, GBNNSS, Waarneming, ISSG
Arthropoda	*Balanus improvisus*	Acorn barnacle	NAm	GB, BE, NTL	EU-Black, DAISIE, NOBANIS, WGITMO, Panov[1]
Arthropoda	*Bemisia tabaci*	Tobacco Whitefly	As	FR, BE, NTL	EU-Black, DAISIE, ISSG
Arthropoda	*Ceratitis capitata*	Mediterranean Fruit-fly	Af	GB, FR, BE, NTL	EU-Black, DAISIE
Arthropoda	*Cercopagis pengoi*	Fish-hook waterflea	As	0	EU-Black, DAISIE, SEBI, NOBANIS, ISSG
Arthropoda	*Chaetogammarus warpachowski*		Eur	0	G&A[3]
Arthropoda	*Charybdis longicollis*	Erythrean swimming crab	Eur	0	EU-Black
Arthropoda	*Chionoecetes opilio*	Queen crab	As	0	NOBANIS
Arthropoda	*Gammarus fasciatus*		NAm	0	Panov[1]
Arthropoda	*Linepithema humile*	Argentine ant	SAm	GB, FR, BE, NTL	EU-Black, DAISIE, SEBI, GBNNSS, ISSG

Arthropoda	*Obesogammarus obesus*		Eur	0	G&A[3]
Arthropoda	*Paralithodes camtschaticus*	Red King Crab	NAm	0	EU-Black, DAISIE, SEBI, NOBANIS, GBNNSS
Arthropoda	*Pontogammarus robustoides*		Eur	0	EU-Black, NOBANIS, G&A[3], Panov[1]
Arthropoda	*Portunus pelagicus*	Blue swimming crab	As	0	EU-Black, DAISIE
Arthropoda	*Thaumetopoeaprocessionea*	Oak Processionary Moth	Eur	GB, FR, BE, NTL	GBNNSS
Arthropoda	*Vespa velutina*	Asian Hornet	As	FR	GBNNSS, Waarneming
Chordata	*Ammotragus lervia*	Barbary sheep	Af	0	EU-Black, SEBI
Chordata	*Aphanius dispar*	Arabian killifish	As	0	EU-Black
Chordata	*Bison bison*	American bison	NAm	0	Nentwing[4]
Chordata	*Boiga irregularis*	Brown tree snake	Au	0	ISSG
Chordata	*Callosciurus finlaysonii*	Finlayson's squirrel	As	0	EU-Black, SEBI, Harmonia, Waarneming, Nentwig[4]
Chordata	*Castor canadensis*	Canadian beaver	NAm	0	EU-Black, SEBI, NOBANIS, GBNNSS, WGITMO
Chordata	*Chrysemys picta*	Painted turtle	NAm	0	EU-Black, GBNNSS
Chordata	*Clarias batrachus*	Walking Catfish	As	0	ISSG
Chordata	*Fistularia commersoni*	Blue-spotted cornetfish	As	0	EU-Black, DAISIE, SEBI
Chordata	*Lates niloticus*	Nile perch	Af	0	ISSG
Chordata	*Muntiacus muntjak*	Indian Muntjac	As	0	Waarneming
Chordata	*Neogobius gymnotrachelus*	Racer goby	Eur	0	G&A[3]
Chordata	*Neogobius melanostomus*	Round Goby	As	BE. NTL	EU-Black, DAISIE, SEBI, NOBANIS, GBNNSS, Harmonia, Waarneming, G&A[2,3]
Chordata	*Proterorhinus marmoratus*	Tubenose Goby	Eur	NTL	GBNNSS, Nentwig[4]
Chordata	*Saurida undosquamis*	True lizardfish	As	0	EU-Black, DAISIE, SEBI
Chordata	*Sciurus carolinensis*	Grey Squirrel	NAm	GB, BE, NTL	EU-Black, DAISIE, SEBI, GBNNSS, Harmonia, Waarneming, ISSG
Chordata	*Seriola fasciata*	Lesser amberjack	NAm	0	EU-Black, SEBI
Chordata	*Siganus rivulatus*	Dusky spinefoot	As	0	EU-Black, DAISIE, SEBI
Dinoflagellata	*Alexandrium catenella*		NAm	GB, FR, NTL	EU-Black, DAISIE, SEBI
Echinodermata	*Asterias amurensis*	Japanese seastar	As	0	ISSG
Mollusca	*Anadara inaequivalvis*	Inequivalve ark	As	0	EU-Black, SEBI

Mollusca	*Dreissena bugensis*	Quagga Mussel	Eur	BE. NTL	EU-Black, G&A[2,3], Panov[1]
Mollusca	*Euglandina rosea*	Cannibal snail	NAm	0	ISSG
Mollusca	*Musculista senhousia*	Asian date mussel	As	FR	EU-Black, DAISIE, SEBI
Mollusca	*Pomacea canaliculata*	Apple snail	SAm	0	ISSG
Mollusca	*Potamocorbula amurensis*	Amur clam	As	0	ISSG
Mollusca	*Theodoxus danubialis*	Danube snail	Eur	0	Panov[1]
Nematoda	*Anguillicola crassus*	Eel swim bladder nematode	As	GB, FR, BE, NTL	EU-Black, DAISIE, SEBI, NOBANIS, GB-NNSS

[1]Panov, V.E., Alexandrov, B., Arbačiauskas, K., Binimelis, R., Copp, G.H., Grabowski, M., Lucy, F., Leuven, R.S.E.W., Nehring, S., Paunović, M., Semenchenko, V. & Son, M.O. (2009) Assessing the risks of aquatic species invasions via european inland waterways: from concepts to environmental indicators. *Integrated Environmental Assessment and Management*, 5, 110-126.

[2]Gallardo, B. & Aldridge, D.C. (2013) The ‘dirty dozen’: socio-economic factors amplify the invasion potential of 12 high risk aquatic invasive species in Great Britain and Ireland. *Journal of Applied Ecology*, 50, 757-766.

[3]Gallardo, B. & Aldridge, D.C. (2013) Priority setting for invasive species management: integrated risk assessment of multiple Ponto Caspian invasive species into Great Britain. *Ecological Applications*, **23**, 352-364.

[4]Nentwig, W., Kuhnel, E. & Bacher, S. (2010) A Generic Impact-Scoring System Applied to Alien Mammals in Europe. *Conservation Biology*, 24, 302-3
