# Supplementary material for: The Importance of the Human Footprint in Shaping the Global Distribution of Terrestrial, Freshwater and Marine Invaders
Source: PLoS One. 2015 May 27;10(5):e0125801. doi: 10.1371/journal.pone.0125801 (PMC4446263; doi:10.1371/journal.pone.0125801)
Supplement: S3 Table — (PDF) [file pone.0125801.s003.pdf]

Table S3. References used to complete the known native and invasive distribution of species.

Species	Reference
Aedes albopictus	Toto J.C., S. Abaga, P. Carnevale, F. Simard. 2003. First report of the oriental mosquito *Aedes albopictus* on the West African island of Bioko, Equatorial Guinea. Medical and Veterinary Entomology, 17(3): 343–346
	Simard F., Nchoutpouen E., Toto J.C., D. Fontenille. 2005. Geographic Distribution and Breeding Site Preference of *Aedes albopictus* and *Aedes aegypti* (Diptera: Culicidae) in Cameroon, Central Africa. Journal of Medical Entomology, 42(5): 726-731
	Medley K.A. 2010. Niche shifts during the global invasion of the Asian tiger mosquito, *Aedes albopictus* Skuse (Culicidae), revealed by reciprocal distribution models. Global Ecology and Biogeography, 19(1): 122–133
Ageratina adenophora	Wang R., Wang Y-Z. 2006. Invasion dynamics and potential spread of the invasive alien plant species Ageratina adenophora (Asteraceae) in China. Diversity and Distribution, 12(4): 1472-4642
Agrilus planipennis	United States Government Accountability Office Report. 2006. Lessons Learned from Three Recent Infestations May Aid in Managing Future Efforts. 118pp.
	Smitley D., McCullough D. How Homeowners Can Protect Ash Trees From the Emerald Ash Borer in Southeastern Michigan. Michigan State University.
	http://www.in.gov/dnr/entomolo/5349.htm
Alexandrium catenella	Hallegraeff, G. M., Bolch, C. J., Blackburn, S. I., Oshima, Y. 1991. Species of the Toxigenic Dinoflagellate Genus *Alexandrium* in Southeastern Australian Waters. Botanica Marina, 34 (6): 575–588
Ammotragus lervia	Cassinello J., Cuzin F., Jdeidi T., Masseti M., Nader I., de Smet K. 2008. *Ammotragus lervia*. In: IUCN 2012. IUCN Red List of Threatened Species. Version 2012.2. www.iucnredlist.org. Downloaded on 06 February 2013.
Anguillicola crassus	Wickström H., P. Clevestam and J. Höglund. 1998. The spreading of *Anguillicola crassus* in freshwater lakes in Sweden. Bull. Fr. Pêche Piscic. 349 : 215-221
	Kirk R. S. 2003. The impact of *Anguillicola crassus* on European eels. Fisheries Management and Ecology, 10(6): 385–394
Anoplolepis gracilipes	http://www.hear.org/species/anoplolepis_gracilipes/
Anoplophora chinensis	Van Der Gaag, D. J., G. Sinatra, P. F. Roversi, A. Loomans, F. Hérard, A. Vukadin. Evaluation of eradication measures against *Anoplophora chinensis* in early stage infestations in Europe. EPPO Bulletin, 40(2): 176–187
	Hérard, F., M. Ciampitti, M. Maspero, H. Krehan, U. Benker, C. Boegel, R. Schrage, L. Bouhot-Delduc, P. Bialooki. 2006. *Anoplophora* species in Europe: infestations and management processes. EPPO Bulletin, 36(3): 470–474
Anoplophora glabripennis	Townsend A. and R. Scachetti-Pereira. 2004. Potential geographic distribution of *Anoplophora glabripennis* (Coleoptera: Cerambycidae) in North America. The American Midland Naturalist, 151(1): 170-178
	Hu J., S. Angeli, S. Schuetz, Y. Luo, A. E. Hajek. 2009. Ecology and management of exotic and endemic Asian longhorned beetle *Anoplophora glabripennis.* Agricultural and Forest Entomology, 11(4): 359–375
Aphanius dispar	Keivany Y., M. Ghorbani. 2012. Distribution of *Aphanius dispar dispar* (Rüppell, 1829) populations in Iran, with a new record from western Iran (Actinopterygii: Cyprinodontidae). Turk J Zool, 36 (6): 824-827
Bemisia tabaci	Lima L.H.C., L. Campos, M.C. Moretzsohn, D. Návia and M.R.V. de Oliveira. 2002. Genetic diversity of *Bemisia tabaci* (Genn.) Populations in Brazil revealed by RAPD markers. Genet. Mol. Biol. 25 (2)
	Skaljac M., K. Zanic, S. G Ban, S. Kontsedalov and M. Ghanim. 2010. Co-infection and localization of secondary symbionts in two whitefly species. BMC Microbiology, 10: 142
	Kairo M.T.K., S.T. Murphy. 2005. Comparative studies on populations of *Pauesia juniperorum* (Hymenoptera: Braconidae), a biological control agent for *Cinara cupressivora* (Hemiptera: Aphididae). Bulletin of Entomological Research, 95(6): 597-

	603
	Tahiri A., A. Sekkat, A. Bennani, M. Granier, G. Delvare, M. Peterschmitt. 2006. Distribution of tomato-infecting begomoviruses and *Bemisia tabaci* biotypes in Morocco. Annals of Applied Biology, 149 (2): 175–186
	Chia-Hung Hsieh, Chung-Hsiung Wang, and Chiun-Cheng Ko. 2006. Analysis of *Bemisia tabaci* (Hemiptera: Aleyrodidae) Species complex and distribution in Eastern Asia based on mitochondrial DNA Markers. Annals of the Entomological Society of America, 99 (5): 768-775
	Xi Teng, Fang-Hao Wan, Dong Chu. 2010. Bemisia tabaci Biotype Q Dominates Other Biotypes Across China. Florida Entomologist, 93(3):363-368
	Bayhan E., Ulusoy M.R., Brown J.K. 2006. Host range, distribution, and natural enemies of Bemisia tabaci 'B biotype' (Hemiptera: Aleyrodidae) in Turkey. Journal of Pest Science, 79(4): 233-240
	Fujiie A., Abdul Mohsen Said Omar, Bahij Sawas A., Abbas A., Abdul Hadi M., Alden Sawas E., Barakat A., Ueda S. Natsuaki K.T. 2009. Geographic distribution of *Bemisia tabaci* biotypes<br>Collected from autumn-cultured potato fields in Syria. J. ISSAAS 15(2): 12-20
Boiga irregularis	Rödder D., Lötters S. 2010. Potential Distribution of the Alien Invasive Brown Tree Snake, *Boiga irregularis* (Reptilia: Colubridae) 1. Pacific Science, 64(1), 11-22<br>www.herpnet2.org
Cabomba caroliniana	Jacobs M.J., Macisaac H.J. 2009. Modelling spread of the invasive macrophyte *Cabomba*<br>*caroliniana*. Freshwater Biology 54: 296–305
Callosciurus finlaysonii	Bertolino S., Genovesi P. 2005. The application of the European strategy on invasive alien species: an example with introduced squirrels. Hystrix, the Italian Journal of Mammalogy, 16(1): 59-69
	Bertolino S., Lurz P.W.W. 2013. *Callosciurus* squirrels: worldwide introductions, ecological impacts and recommendations to prevent the establishment of new invasive populations. Mammal Review, 43: 22-33
Castor canadensis	Skewes O., Gonzalez F., Olave R., Ávila A., Vargas V., Paulsen P., König H. 2006. Abundance and distribution of American beaver, *Castor canadensis* (Kuhl 1820), in Tierra del Fuego and Navarino islands, Chile. European Journal of Wildlife Research, 52: 292-296
Ceratitis capitata	Meyer M. De, M. P. Robertson, A. T. Peterson, M. W. Mansell. 2008. Ecological niches and potential geographical distributions of Mediterranean fruit fly (*Ceratitis capitata*) and Natal fruit fly (*Ceratitis rosa*). Journal of Biogeography, 35(2): 270–281
	Vera M.T., Rodriguez R., Segura D.F., Cladera J.L., Sutherst R.W. 2002. Potential Geographical Distribution of the Mediterranean Fruit Fly, Ceratitis capitata (Diptera: Tephritidae), with Emphasis on Argentina and Australia. Environ. Entomol. 31(6): 1009-1022
	Oroño, L. E., Albornoz-Medina, P., Núñez-Campero, S., Van Nieuwenhove, G. A., Bezdjian, L. P., Martin, C. B., Ovruski, S. M. 2006. Update of host plant list of Anastrepha fraterculus and Ceratitis capitata in Argentina. In R. L. Sugayama, R. A. Zucchi, S. M. Ovruski, & J. Sivinski (Eds.), Proceedings of the 7th International Symposium on Fruit Flies of Economic Importance (pp. 10-15).
Charybdis longicollis	Innocenti G., Bella S. G. 2007. Modus vivendi: Invasive host/parasite relations—*Charybdis longicollis* Leene, 1938 (Brachyura: Portunidae) and *Heterosaccus dollfusi* Boschma, 1960 (Rhizocephala: Sacculinidae). Hydrobiologia, 590: 95-101
	Lewinsohn C., Lipke B.H. 1964. New records of decapod Crustacea from the Mediterranean coast of Israel and the eastern Mediterranean. Zoologische Mededelingen 40(8): 45-63
	Ozcan T., Katagan T., Kocatas A. 2005. Brachyuran crabs from Iskenderun Bay (southeastern Turkey). Crustaceana, 237-243
Euglandina rosea	South Pacific Regional Environment Programme. 2000. Invasive species in the Pacific: A technical review and draft regional strategy. 197 pp
	Meyer III, W. M., & Cowie, R. H. 2011. Distribution, movement, and microhabitat use of the introduced predatory snail Euglandina rosea in Hawaii: implications for management. Invertebrate Biology, 130(4): 325–333
Linepithema	Holway D.A. 1995. Distribution of the Argentine Ant (*Linepithema humile*) in Northern

humile	California. Conservation Biology, 9(6): 1634-1637
	Roura-Pascual N., A. V. Suarez, C. Gómez, P. Pons, Y. Touyama, A.L. Wild, and A.T. Peterson. 2004. Geographical potential of Argentine ants (*Linepithema humile* Mayr) in the face of global climate change. Proc. R. Soc. Lond. B 271(1557): 2527-2535
Liriomyza huidobrensis	He, L., Zhang, Y., Xiao, N., Wei, J., & Kuang, R. 2003. Liriomyza huidobrensis in Yunnan, China: current distribution and genetic structure of a recently established population. Entomologia experimentalis et applicata, 102(3): 213-219
Marmokrebs	D.M. Holdich, J.D. Reynolds, C. Souty-Grosset and P.J. Sibley. 2009. A review of the ever increasing threat to European crayfish from non-indigenous crayfish species. Knowl. Managt. Aquatic Ecosyst. 11: 394-395
	Martin P., Nathan J. Dorn, Tadashi Kawai, Craig van der Heiden, Gerhard Scholtz. 2010. The enigmatic Marmorkrebs (marbled crayfish) is the parthenogenetic form of Procambarus fallax (Hagen, 1870). Contributions to Zoology, 79 (3)
	Nonnis Marzano F., M. Scalici, S. Chiesa, F. Gherardi, A. Piccinini and G. Gibertini. 2009. The first record of the marbled crayfish adds further threats to fresh waters in Italy. Aquatic Invasions, 4(2): 401-404
	Hendrix, A. N., & Loftus, W. F. (2000). Distribution and relative abundance of the crayfishes Procambarus alleni (Faxon) and P. fallax (Hagen) in southern Florida. Wetlands, 20(1), 194-199.
	Feria, T. P., & Faulkes, Z. (2011). Forecasting the distribution of Marmorkrebs, a parthenogenetic crayfish with high invasive potential, in Madagascar, Europe, and North America. Aquatic Invasions, 6(1), 55-67.
Miconia calvescens	Department of primary industries and Fisheries. Queensland Government. 2006. Pest plant risk assessment. 14 pp.
Musculista senhousia	Eun Jung Choya, Soonmo Anb, Chang-Keun Kang. 2007. Pathways of organic matter through food webs of diverse habitats in the regulated Nakdong River estuary (Korea). Estuarine, Coastal and Shelf Science, 78(1): 215–226
	Bacheleta G., H. Blancheta, M. Cotteta, C. Danga, X. de Montaudouina, A. de Moura Queirósa, B. Gouillieuxa and N. Lavesque. 2009. A round-the-world tour almost completed: first records of the invasive mussel *Musculista senhousia* in the north-east Atlantic (southern Bay of Biscay). Marine Biodiversity Records, 2, e119
	Zhoua H., Z.N. Zhanga, X.S. Liua, L.H. Tuc, Z.S. Yua. 2007. Changes in the shelf macrobenthic community over large temporal and spatial scales in the Bohai Sea, China. Journal of Marine Systems, 67(3–4) 312–321
Nyctereudes procynoides	http://www.canids.org/species/Nyctereutes_procyonoides.htm
Phytophthora cinnamomi	Robin C., M.L. Desprez-Loustau, G. Capron and C. Delatour. 1998. First record of *Phytophthora cinnamomi* on cork and holm oaks in France and evidence of pathogenicity. Ann. For. Sci. 55 (8) 869-883
	http://www.scientificsocieties.org/aps/proceedings/sod/Papers/Brasier/default.htm
Pomacea canaliculata	Lv S., Zhang Y., Liu H.X., Hu L., Yang K., Steinmann P., Chen Z., Wang L.Y., Utzinger J., Zhou X.N. 2009. Invasive Snails and an Emerging Infectious Disease: Results from the First National Survey on *Angiostrongylus cantonensis* in China. PLoS Neglected Tropical Disease, 3(2): e368
	Seuffert, M. E., & Martín, P. R. (2012). Distribution of the apple snail< i> Pomacea canaliculata</i> in Pampean streams (Argentina) at different spatial scales. Limnologica-Ecology and Management of Inland Waters.
	Kenji, I. (2003). Expansion of the golden apple snail, Pomacea canaliculata, and features of its habitat. National Agricultural Research Center". Department of Entomology and Nematology. National Agricultural Research Center Kannondai, 3-1.
Rhopilema nomadica	Deidun A., S. Arrigo and S. Piraino. 2011. The westernmost record of *Rhopilema nomadica* (Galil, 1990) in the Mediterranean – off the Maltese Islands. Aquatic Invasions, Suppl. 99-103
	Gülşahin, N., & Tarkan, A. N. (2011). The first confirmed record of the alien jellyfish Rhopilema nomadica Galil, 1990 from the southern Aegean coast of Turkey. Aquatic Invasions, 6(1), S95-S97.
	Siokou-Frangou, I., Sarantakos, K., & Christou, E. D. (2006). First record of the scyphomedusa Rhopilema nomadica Galil, 1990 (Cnidaria: Scyphozoa: Rhizostomeae) in Greece. Aquatic Invasions, 1(3), 194-195.

Seiridium cardinale	Xenopoulos S., S. Diamandis. 1985. A distribution map for *Seiridium cardinale* causing the cypress canker disease in Greece. European Journal of Forest Pathology, 15(4): 223–226
	Tsopelas P., I. Barnes, M. J. Wingfield, S. Xenopoulos. 2007. *Seiridium cardinale* on Juniperus species in Greece. Forest Pathology, 37 (5): 338–347
	Zoccaa A., C. Zaninia, A. Aimia, G. Frigimelicaa, N. La Porta, A. Battistia. 2008. Spread of plant pathogens and insect vectors at the northern range margin of cypress in Italy. Acta Oecologica, 33 (3): 307–313
	Moricca S., I. Børja, G. G. Vendramin, P. Raddi. 2000. Differentiation of *Seiridium* species associated with virulent cankers on cypress in the Mediterranean region by PCR-SSCP. Plant Pathology, 49(6): 774–781
Seriola fasciata	Andaloro F., Falautano, M., Sinopoli, M., Passarelli, F. M., Pipitone, C., Addis, Castriota, L. 2005. The lesser amberjack *Seriola fasciata* (Perciformes: Carangidae) in the Mediterranean: A recent colonist. Cybium 29(2): 141-145
Thaumetopoea processionaria	Groenen F., Meurisse N. 2011. Historical distribution of the oak processionary moth *Thaumetopoea processionea* in Europe suggests recolonization instead of expansion. Agricultural and Forest Entomology, 14(2): 147-155
Undaria pinnatifida	Zabin C. J., Ashton G. V., Brown C. W., Ruiz G. M. 2009. Northern range expansion of the Asian kelp *Undaria pinnatifida* (Harvey) Suringar (Laminariales, Phaeophyceae) in western North America. Aquatic Invasions 4(3): 429-434
	Meretta P. E., Matula C. V., Casas G. 2012. Occurrence of the alien kelp *Undaria pinnatifida* (Laminariales, Phaeophyceae) in Mar del Plata, Argentina. BioInvasions Records 1: 59-63
	Russell L. K., Hepburn C. D., Hurd C. L., Stuart M. D. 2008. The expanding range of *Undaria pinnatifida* in southern New Zealand: distribution, dispersal mechanisms and the invasion of wave-exposed environments. Biological Invasions 10(1): 103-115
