# Supplementary material for: The Importance of the Human Footprint in Shaping the Global Distribution of Terrestrial, Freshwater and Marine Invaders
Source: PLoS One. 2015 May 27;10(5):e0125801. doi: 10.1371/journal.pone.0125801 (PMC4446263; doi:10.1371/journal.pone.0125801)
Supplement: S4 Table — (PDF) [file pone.0125801.s004.pdf]

Table S4. Correlation between continental layers used for calibrating Species Distribution Models. Annual T: annual mean temperature, T seasonality: temperature annual seasonality, Max T: mean temperature in the warmest month, Min T: mean temperature in the coldest month, Annual PP: annual precipitation, PP driest: mean precipitation of the driest month, PP seasonality: precipitation annual seasonality, HII: Human Influence Index. Note: categorical variables such as geology and land cover are not included in this table. Pearson moment correlations measured using ENMTools v.1.3. Land-use not included here because it is categorical.

	**Altitude**	**Annual T**	**T seasonality**	**Max T**	**Min T**	**Annual PP**	**PP driest**	**PP seasonality**	**HII**	**Population Density**	**Port proximity**	**Road proximity**
**Altitude**	1.00											
**Annual T**	-0.10	1.00										
**T seasonality**	-0.07	-0.78	1.00									
**Max T**	-0.19	0.80	-0.56	1.00								
**Min T**	-0.09	0.80	-0.95	0.80	1.00							
**Annual PP**	-0.12	0.40	-0.55	0.13	0.50	1.00						
**PP driest**	-0.15	0.11	-0.24	-0.08	0.20	0.70	1.00					
**PP seasonality**	0.24	0.31	-0.19	0.37	0.23	-0.18	-0.51	1.00				
**HII**	-0.05	0.33	-0.31	0.25	0.35	0.24	0.11	0.00	1.00			
**Pop. Density**	-0.02	0.05	-0.04	0.04	0.05	0.04	0.00	0.03	0.18	1.00		
**Port proximity**	-0.05	-0.53	0.53	-0.40	-0.54	-0.23	-0.17	0.00	-0.45	-0.06	1.00	
**Road proximity**	-0.15	-0.57	0.43	-0.57	-0.51	-0.19	-0.05	-0.11	-0.39	-0.04	0.58	1.00
