# Supplementary material for: The Importance of the Human Footprint in Shaping the Global Distribution of Terrestrial, Freshwater and Marine Invaders
Source: PLoS One. 2015 May 27;10(5):e0125801. doi: 10.1371/journal.pone.0125801 (PMC4446263; doi:10.1371/journal.pone.0125801)
Supplement: S5 Table — (PDF) [file pone.0125801.s005.pdf]

Table S5. Correlation between human footprint variables and the sampling effort map. HII: Human Influence Index. Pearson moment correlations measured using ENMTools v.1.3.

	**Sampling effort**	**Road proximity**	**HII**	**Pop. Density**	**Port proximity**
**Sampling effort**	1.00	-0.08	0.21	0.04	-0.18
**Road proximity**		1.00	-0.39	-0.04	0.58
**HII**			1.00	0.18	-0.45
**Pop. Density**				1.00	-0.06
**Port proximity**					1.00
