# Supplementary material for: The Importance of the Human Footprint in Shaping the Global Distribution of Terrestrial, Freshwater and Marine Invaders
Source: PLoS One. 2015 May 27;10(5):e0125801. doi: 10.1371/journal.pone.0125801 (PMC4446263; doi:10.1371/journal.pone.0125801)
Supplement: S6 Table — (PDF) [file pone.0125801.s006.pdf]

Table S6. Correlation between marine layers used for calibrating Species Distribution Models. PAR: photosynthetic active radiation. Pearson moment correlations measured using ENMTools v.1.3.

	**Calcite**	**Max Chlorophyll**	**Min Chlorophyll**	**Dissolved Oxygen**	**PAR**	**Nitrate**	**Phosphate**	**Salinity**	**Silicate**	**Max Surface Temperature**
**Calcite**	1.00									
**Max Chlorophyll**	0.40	1.00								
**Min Chlorophyll**	0.48	0.74	1.00							
**Dissolved Oxygen**	0.07	0.17	0.13	1.00						
**PAR**	-0.05	-0.10	-0.09	-0.81	1.00					
**phosphate**	-0.16	-0.22	-0.25	-0.41	0.39	1.00				
**phosphate**	-0.02	0.02	-0.01	0.70	-0.60	-0.44	1.00			
**Salinity**	-0.24	-0.47	-0.44	-0.49	0.41	0.30	-0.15	1.00		
**Silicate**	0.01	0.07	0.03	0.64	-0.58	-0.41	0.83	-0.18	1.00	
**Max Temperature**	-0.03	-0.11	-0.09	-0.97	0.79	0.42	-0.79	0.38	-0.70	1.00
