# Supplementary material for: The Importance of the Human Footprint in Shaping the Global Distribution of Terrestrial, Freshwater and Marine Invaders
Source: PLoS One. 2015 May 27;10(5):e0125801. doi: 10.1371/journal.pone.0125801 (PMC4446263; doi:10.1371/journal.pone.0125801)
Supplement: S7 Table — (PDF) [file pone.0125801.s007.pdf]

Table S7. Species Distribution Model (SDM) output statistics for terrestrial plants, terrestrial animals, and aquatic inland species. Gaps in variable permutation importance represent variables dropped during model optimization.

	Training samples	Regularized training gain	Unregularized training gain	Test samples	Test gain	Test AUC	Variable Permutation Importance														Entropy	MaxTSS	Sensitivity
							Altitude	Annual PP	PP Driest Month	PP seasonality	Annual Mean T	T Seasonality	Maximum Temperature	Minimum T	Geology	HII	Land-cover	Population Density	Portsproximity	Road proximity			
**Terrestrial Plants**																							
A.dealbata	607	2.77	2.98	261	3.00	0.98	1.7	2.5	1.1	2.7	45.6	35.7	3.8	1.8	0.1	2.1	1.3	0.2	1.3		6.5	0.10	0.98
C.edulis	298	3.26	3.49	129	3.10	0.98		0.1	1.1	1.0	17.5	4.8	1.5	69.2	0.0	1.7		0.1	3.1	0.0	6.0	0.04	0.95
C.selloana	362	2.92	3.16	156	2.83	0.97	2.9	9.1	1.2	0.4	20.1	23.5	10.3	9.7	0.0	16.3	1.0	1.7	2.7	1.1	6.3	0.10	0.92
E.lobata	842	2.30	2.47	361	2.29	0.96	1.4	3.5	6.7	3.6	41.8	4.0	1.3	20.7	1.3	5.4	1.5		3.3	5.5	7.0	0.27	0.92
H.gardnerianum	30	2.27	3.64	14	2.43	0.93	1.9	0.0	0.0	0.0	0.0	23.7	0.0	20.8	0.0	0.2	1.2	0.0	17.2	34.9	6.9	0.20	0.80
I.cylindrica	1336	1.84	2.06	573	1.84	0.94	2.8	3.7	1.8	4.9	56.8	11.0	9.0	0.4	0.2	2.0	2.8	0.3	3.9		7.6	0.20	0.88
L.camara	1446	1.73	1.87	620	1.79	0.94	0.8	2.9	13.2	2.1	19.9	27.3	0.8	5.4	1.0	19.9	1.2	0.5	4.9	2.1	6.4	0.18	0.95
M.calvescens	184	2.78	3.25	80	3.13	0.98	0.0	0.1	0.3	0.4	1.4	76.9	0.4	6.9	0.5	2.1	3.3	5.7	5.7	2.7	7.0	0.14	0.91
M.micrantha	539	2.21	2.44	232	2.17	0.96	3.3	2.0	2.7	0.6	2.8	9.7	0.0	63.0	0.1	5.5	2.0	0.9	4.7		5.8	0.13	0.94
M.quinquenervia	273	3.43	3.77	118	3.60	0.99	14.8	3.4	9.0	12.9	13.0	4.0	22.5		0.5	7.5		4.0	8.4	0.7	6.8	0.14	0.93
O.ficusindica	181	2.67	3.07	78	2.81	0.97	0.3	0.8	1.0	1.1	28.0	4.4	0.0	60.2	0.4	0.9	0.2	0.6	1.9		6.6	0.13	0.91
O.pescaprae	298	3.26	3.49	128	3.14	0.98	0.2	1.2	10.1	0.8	33.1	6.9	0.2	28.3	0.6	15.7	0.4	0.8	1.7		6.0	0.11	0.93
P.glandulosa	368	2.43	2.76	159	2.47	0.97	2.9	6.4		4.0	32.1	5.6	2.8	18.5	0.5	13.1	3.5	3.9	5.9	5.8	6.5	0.15	0.93
P.montana	521	2.74	2.93	224	2.72	0.97	0.9	56.4	3.3	2.7	7.7	12.5	1.1	0.5	0.2	2.2	4.5	0.2	2.1	57.0	6.9	0.13	0.94
R.ellipticus	55	2.27	3.54	24	3.10	0.98	5.9	4.2	2.4	0.4	23.6	2.1	0.0		0.3	0.0	0.2	0.0	3.8	0.8	6.6	0.56	0.78
S.terebinthifolius	130	2.62	3.25	56	2.95	0.97	3.7	0.5	0.7	0.0	17.6	65.8	0.0		0.4		0.0		10.3		8.1	0.13	0.90
T.ramosissima	455	1.14	1.39	196	1.30	0.90	19.8					47.3			0.7				32.2	0.6	7.4	0.27	0.91
**Terrestrial Animals**																							
A.albopictus	996	1.75	1.92	428	1.72	0.93	0.7	31.1		16.0	4.2	9.1	6.7	6.6	1.0	1.1	4.8	0.9	13.6	4.3	7.5	0.20	0.93
A.glabripennis	40	1.99	2.88	18	2.32	0.96		10.1	0.0		0.0	41.3	24.6		1.3	0.1			22.5		7.2	0.37	0.88
A.gracilipes	183	3.07	3.35	79	2.90	0.98	0.4	0.8				4.9	5.6	79.9	0.3	5.6	0.1	0.1	0.3	1.9	6.2	0.04	0.96
A.lervia	137	2.59	3.29	59	3.04	0.98	3.9	4.5	4.8	0.0	26.2	27.7	6.2	24.4		0.2		0.2	2.0		6.6	0.19	0.92
B.bison	98	1.85	2.46	43	2.26	0.94	18.3		31.0			10.3	13.3	7.6		4.3	3.8	2.6	5.8	2.9	7.4	0.14	0.94

B.irregularis	335	2.72	3.21	144	2.89	0.98	12.6	32.0		2.6	16.4	8.2	9.9	0.3	1.4	2.7	2.5		9.3	2.1	6.6	0.16	0.93
B.tabaci	242	2.04	2.35	104	2.02	0.94	0.0	0.5	0.1	3.3	3.3	10.8	3.0	61.6	0.6	10.6		0.0	3.9	2.1	7.2	0.11	0.93
C.canadensis	427	1.76	2.11	183	1.88	0.94	1.4	0.7	0.8	8.6		6.2	10.1	35.5	0.4	8.6	7.4	3.9	15.2	1.1	7.5	0.23	0.93
C.capitata	385	1.89	2.20	166	1.92	0.94	0.2	10.7	5.3	1.9	0.5	16.5	12.6	21.0	0.2	23.7	1.4	2.3	3.6		7.3	0.24	0.88
C.finlaysonii	19	0.79	1.61	9	1.66	0.97		0.0	0.0	0.0		0.0		32.9	20.4	0.0		0.0	18.3	28.4	8.4	0.62	0.86
C.picta	645	2.39	2.65	277	2.49	0.97	2.6	3.9	2.0	6.3	0.0	3.0	29.0	25.7		4.1	2.9	0.2	17.1	3.3	6.9	0.15	0.94
E.rosea	62	2.67	3.72	27	2.85	0.93	3.1		16.9		63.9	0.0	0.0	0.3	0.1	12.1		0.0		3.5	6.5	0.20	0.81
L.humile	567	2.61	2.82	243	2.59	0.97	0.5		2.0	1.5	23.1	32.6	0.8		0.6	28.0	1.6		5.7	3.6	6.6	0.16	0.90
M.muntjak	28	2.06	2.69	13	1.97	0.95	1.5	2.2	0.0	3.6		65.2	0.0	0.0		11.1	6.4		6.7	3.3	7.2	0.37	0.73
M.neglecta	143	3.92	4.19	62	3.96	0.99	46.9	0.0	1.8	0.0	0.0	13.7	0.1	23.0			1.3	0.1	13.0		5.3	0.11	0.97
P.canaliculata	112	1.80	2.31	49	2.27	0.95	0.0	14.6	8.4	0.3			0.6	70.4	0.2	0.0			4.2	1.3	7.4	0.19	0.89
S.carolinensis	578	2.99	3.16	248	2.89	0.98	9.9	7.9	20.1		5.1	5.1	3.7	22.2	1.7	5.0		1.2	18.2		6.3	0.09	0.96
T.processionea	267	3.24	3.52	115	3.10	0.98	5.6	7.0	7.6		11.9	35.7	9.3			14.4		0.8	6.5	1.4	6.0	0.13	0.91
V.velutina	289	3.65	3.90	125	3.59	0.99	1.4	28.6	1.1	22.1	0.1	37.5	1.1	3.8	0.5	3.0	0.7	0.0		0.0	5.6	0.16	0.92
Aquatic Inland organisms																							
A.crassus	41	2.63	3.46	18	2.92	0.98	3.8	0.0	0.1	0.0	0.7	0.0	0.0	8.1	0.0	0.0	0.8		80.7	5.9	35.7	0.45	0.80
A.dispar	75	3.86	4.32	33	3.66	0.99	0.0	29.8		0.6		4.6	0.0	23.0		2.4			39.5	0.1	5.4	0.17	0.87
C.batrachus	145	4.12	4.41	63	3.68	0.98	2.9	13.7	0.0	1.6	33.2	2.2			1.9	0.1	0.0	0.1	44.0	0.3	5.1	0.03	0.90
C.caroliniana	322	2.95	3.18	138	2.92	0.98	24.5	11.6		23.1	2.1	12.0	9.7	13.1	0.5	1.5			1.3	0.6	6.3	0.10	0.92
C.pengoi	110	3.86	4.14	48	4.03	0.99	9.1			1.4	36.3	20.7		30.5	0.8		1.2	0.0			5.4	0.04	0.99
C.warpachowski	54	2.92	3.47	24	3.60	0.99	30.3	0.0	4.9	9.2	9.0	7.1	0.0	22.4		0.3	0.1			16.8	6.3	0.23	1.00
D.bugensis	251	2.78	3.12	108	3.01	0.98	8.3	0.4	2.8	44.9	13.2	9.3	0.2		1.1	4.2	0.7		14.9		6.5	0.12	0.95
E.crassipes	956	2.02	2.20	411	2.06	0.95	7.8	4.9	2.0	1.7	20.7	10.6	12.5	1.1		11.9	0.9	2.3	6.1	17.5	7.2	0.17	0.90
G.fasciatus	42	2.60	3.54	19	2.67	0.96		1.5		25.1		6.6			1.0	7.4			58.4		6.6	0.16	0.84
L.niloticus	40	1.48	2.37	18	2.49	0.94	2.0	2.7		3.7	80.1				1.1	3.8	4.2	2.6		0.0	7.7	0.31	0.86
M.heterophyllum	445	2.08	2.35	191	2.28	0.96	10.8			33.3			19.4				2.5		34.0		7.2	0.22	0.92
N.gymnotrachelus	24	2.66	3.30	11	3.01	0.98	37.5	2.6	0.0		19.3	0.2	0.0	36.2		0.1			4.0		6.6	0.31	0.94
N.melanostomus	281	3.45	3.75	121	3.33	0.98	20.0	20.5	7.4	23.1		18.0	5.1	0.1		1.8			4.1		5.8	0.08	0.95
O.obesus	59	2.69	3.34	26	3.44	0.99	2.8	15.1	12.7	6.5		5.9		53.2	0.6	2.4	0.9				6.5	0.09	1.00
P.marmoratus	47	3.35	3.90	21	3.27	0.98	0.5	3.2	0.0	46.4	0.2	0.0	0.0	31.8	1.1	2.9	1.8	0.0	11.9	0.2	5.9	0.10	0.90
P.robustoides	123	3.43	3.85	54	3.57	0.99	13.7	0.2	4.2	3.1	15.4	6.7	0.0	53.2	0.0	0.3	0.6		2.0	0.6	5.8	0.06	0.95
T.danubialis	24	2.75	3.48	11	2.89	0.98		0.0	0.0		21.8		1.3	53.3	2.7	0.0	0.3	0.0	5.4	15.3	6.5	0.44	0.83

DEFINITION OF TERMS (modified from MaxEnt's tutorial in http://www.cs.princeton.edu/~schapire/maxent/):
Training samples: number of presence records used for training the model (70% of the total)
Regularized training gain: measure of goodness of fit of models. It represents the presence likelihood of training records in comparison with background records. Gain is regularized for the number of terms in the model to avoid overfitting
Unregularized training gain: model gain not compensating for the number of predictors in the model
Test samples: number of presence records used for testing the model (30% of the total)
Test gain: model gain calculated from presence records held out to test the model
Test AUC: Area Under the ROC curve calculated using test data. A model that performs no better than random will have a test AUC of 0.5 whereas a model with perfect discrimination will score 1
Variable permutation importance: % the variable contributed to the final model. Blanks identify variables that were dropped during model optimization (backwards stepwise selection)
Entropy: Measure of the disorder in the species distribution
MaxTSS: Maximum training sensitivity plus specificity threshold used for transforming continuous (0-100%) into binary (0-1) predictions
Sensitivity: Percentage of occurrence records correctly classified by the (thresholded) predictions
