# Supplementary material for: The Importance of the Human Footprint in Shaping the Global Distribution of Terrestrial, Freshwater and Marine Invaders
Source: PLoS One. 2015 May 27;10(5):e0125801. doi: 10.1371/journal.pone.0125801 (PMC4446263; doi:10.1371/journal.pone.0125801)
Supplement: S8 Table — (PDF) [file pone.0125801.s008.pdf]

Table S8. Species Distribution Model (SDM) output statistics for marine invaders. See definition of terms in Table S3. Gaps in variable permutation importance represent variables dropped during model optimization.

							Variable Permutation Importance															
	**Training samples**	**Regularized training gain**	**Unregularized training**	**Test samples**	**Test gain**	**Test AUC**	**Calcite**	**Maximum chlorophyll-a**	**Minimum Chlorophyll-a**	**Dissolved Oxygen**	**Nitrate**	**Maximum PAR**	**pH**	**Phosphate**	**Salinity**	**Silicate**	**Max Surface Sea T**	**Min Surface Sea T**	**MHI**	**Entropy**	**MaxTSS**	**Sensitivity**
A.amurensis	65	4.18	4.68	29	4.06	0.99	0.7	0.0	1.6	2.5	40.7	0.8	0.0	3.7	1.0	1.9	2.1	45.1		5.1	0.12	0.86
A.catenella	70	1.88	2.86	31	2.96	0.99	0.6	31.5	1.1	17.5		0.0	0.0	2.5	1.8		3.8	41.1		7.3	0.28	0.98
A.inaequivalvis	32	3.01	4.21	14	3.55	0.98	0.0	0.9	0.0	0.0	47.6	0.0	2.0	0.0	0.6	13.9	30.8	4.1		6.2	0.31	0.87
A.taxiformis	106	2.22	2.91	46	2.56	0.96	7.7	1.5	8.4	0.0	43.5	8.9	3.2	0.0	1.2	0.3	1.2	21.6	2.5	7.0	0.26	0.78
B.improvisus	254	3.63	3.84	109	3.81	0.99	12.2	0.2	4.2	4.6	11.9		0.6	0.9	11.1	13.6	40.4		0.3	5.6	0.04	0.95
C.fragile	455	2.93	3.19	195	2.91	0.98	1.6	6.6	64.9	9.4	4.4		0.7		3.8		1.9	6.5	0.1	6.3	0.08	0.94
C.longicollis	32	3.87	4.85	14	4.75	0.99		5.7	0.0	0.0	18.4	39.3	0.0		0.0	0.0	11.4	25.3	0.0	5.3	0.23	0.87
C.opilio	951	2.94	3.01	408	2.99	0.98	0.5	18.7	1.4	6.8	7.2		0.7	29.5	9.8	3.4	15.7	5.4	1.0	6.3	0.14	0.98
C.taxifolia	182	2.58	3.03	78	2.84	0.97	65.7		0.0	0.0	17.0	5.9	1.5		0.9	1.3	7.3		0.4	6.6	0.15	0.90
C.wailesii	873	2.62	2.82	375	2.60	0.97	1.4	49.0	1.7	2.7	2.3	3.5	0.5	4.7	6.0	1.0	18.8	8.3	0.2	6.6	0.19	0.93
F.commersonii	604	2.11	2.43	260	2.11	0.95	13.6		5.2	18.8	17.4	6.9	2.5	4.3	3.3	1.5		25.9	0.5	7.1	0.23	0.82
M.senhousia	78	3.80	4.42	34	3.73	0.99	2.7	10.0		0.9	50.2	11.8	0.0	0.0	2.8	6.3	0.8	14.1	0.5	5.4	0.02	0.96
P.amurensis	22	3.35	4.93	10	4.47	1.00	0.0	0.0	0.0	0.1	19.6	10.3			0.3	24.3	0.0	45.1	0.3	5.8	0.30	0.94
P.camtschaticus	503	3.27	3.43	216	3.29	0.99	1.1	15.8	6.0	1.9	8.8	2.8	0.3	10.4	6.2	2.7	37.7	6.3	0.0	6.0	0.10	0.97
P.pelagicus	148	3.10	3.47	64	3.28	0.99	0.3	5.9	4.4	0.7	74.6	10.2	1.2		0.1	1.5	0.5	0.6	0.0	6.1	0.14	0.93
S.fasciata	71	2.57	3.25	31	3.08	0.97	2.9		1.1	0.0	21.2	0.9	0.9	1.5	0.0	26.0	38.7	6.3	0.4	6.6	0.07	0.95
S.rivulatus	301	3.75	3.97	129	3.76	0.99	0.3	42.9	1.0	0.3	45.4	2.6	2.7		2.9	0.7	0.0	1.2	0.1	5.5	0.04	0.98
S.undosquamis	958	2.63	2.75	411	2.55	0.97	1.0	30.1	2.2	0.4	41.0	10.5	1.8	1.1	0.1	5.4	1.5	4.8	0.0	6.6	0.14	0.93
U.pinnatifida	95	3.42	3.95	42	3.87	0.99	0.0	0.3	61.1	2.3	0.0	0.0	0.1	6.0	0.7	0.1	2.1	27.1	0.4	5.8	0.07	1.00

DEFINITION OF TERMS (modified from MaxEnt's tutorial in http://www.cs.princeton.edu/~schapire/maxent/):

Training samples: number of presence records used for training the model (70% of the total)

Regularized training gain: measure of goodness of fit of models. It represents the presence likelihood of training records in comparison with background records. Gain is regularized for the number of terms in the model to avoid overfitting

Unregularized training gain: model gain not compensating for the number of predictors in the model

Test samples: number of presence records used for testing the model (30% of the total)

Test gain: model gain calculated from presence records held out to test the model

Test AUC: Area Under the ROC curve calculated using test data. A model that performs no better than random will have a test AUC of 0.5 whereas a model with perfect discrimination will score 1

Variable permutation importance: % the variable contributed to the final model. Blanks identify variables that were dropped during model optimization (backwards stepwise selection)

Entropy: Measure of the disorder in the species distribution

MaxTSS: Maximum training sensitivity plus specificity threshold used for transforming continuous (0-100%) into binary (0-1) predictions

Sensitivity: Percentage of occurrence records correctly classified by the (thresholded) predictions
