# Supplementary material for: The Importance of the Human Footprint in Shaping the Global Distribution of Terrestrial, Freshwater and Marine Invaders
Source: PLoS One. 2015 May 27;10(5):e0125801. doi: 10.1371/journal.pone.0125801 (PMC4446263; doi:10.1371/journal.pone.0125801)

Figure S1. Sampling effort map generated to investigate the species-people correlation. The map was generated by combining the occurrence records for the 72 species investigated.

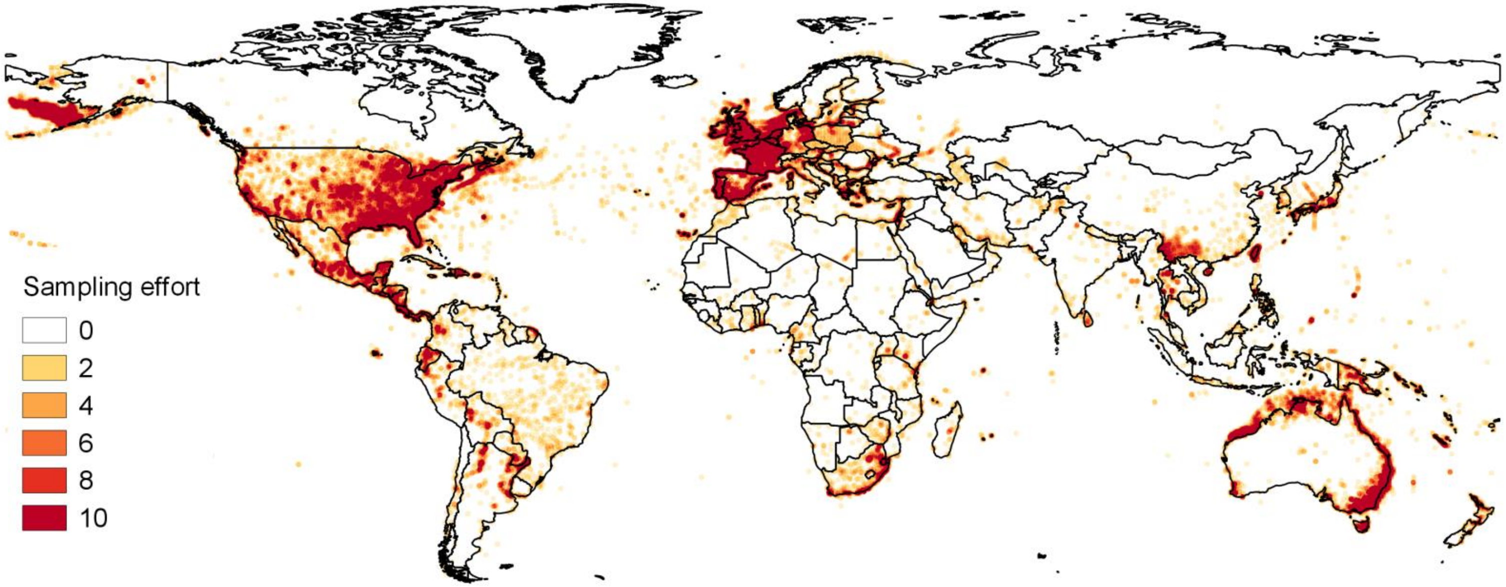

Supplement: S1 Fig — (PDF) [file pone.0125801.s009.pdf]
