# Supplementary material for: The Importance of the Human Footprint in Shaping the Global Distribution of Terrestrial, Freshwater and Marine Invaders
Source: PLoS One. 2015 May 27;10(5):e0125801. doi: 10.1371/journal.pone.0125801 (PMC4446263; doi:10.1371/journal.pone.0125801)

Figure S2. Map of global Influence Index (HII). In red, areas with HII>25, which according to our models generally lead to suitability scores for invasion higher than 50%.

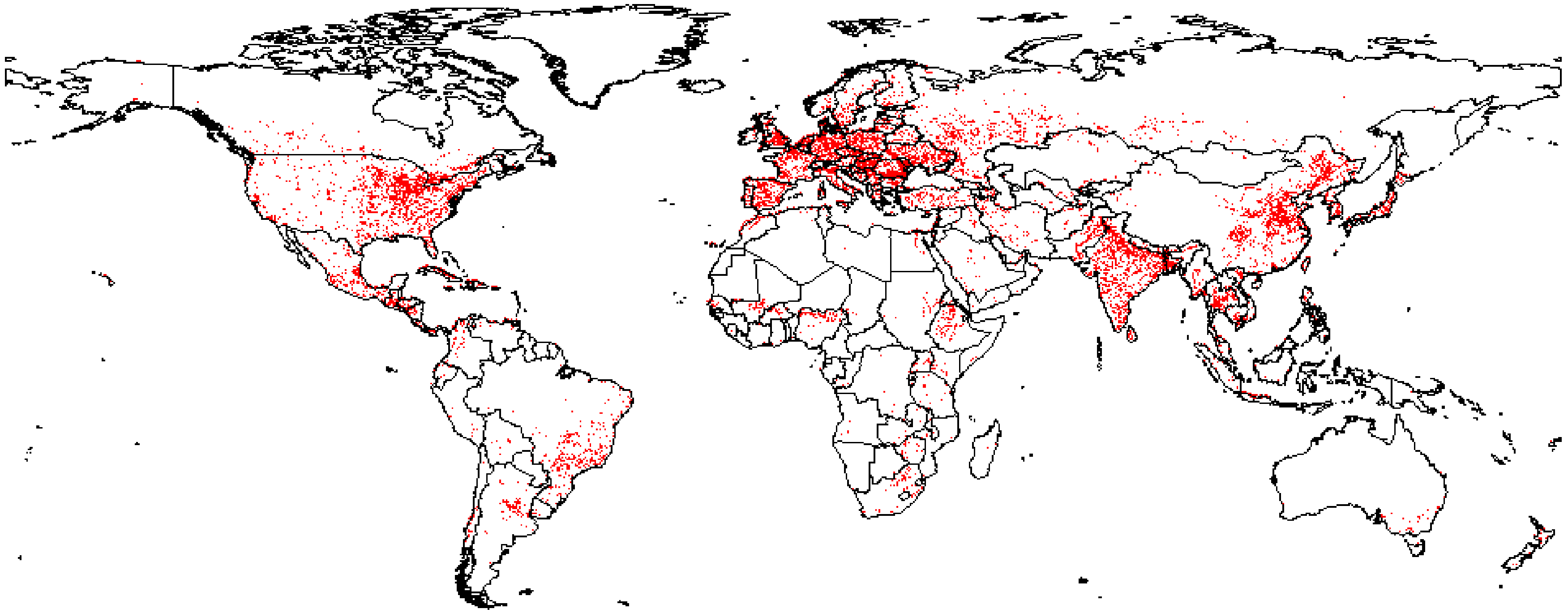

Supplement: S2 Fig — (PDF) [file pone.0125801.s010.pdf]
